# The Biosynthesis of Enzymatically Oxidized Lipids

**DOI:** 10.3389/fendo.2020.591819

**Published:** 2020-11-19

**Authors:** Ali A. Hajeyah, William J. Griffiths, Yuqin Wang, Andrew J. Finch, Valerie B. O’Donnell

**Affiliations:** ^1^Systems Immunity Research Institute and Division of Infection and Immunity, Cardiff University, Cardiff, United Kingdom; ^2^Institute of Life Science, Swansea University Medical School, Swansea, United Kingdom; ^3^Centre for Tumour Biology, Barts Cancer Institute, Queen Mary University of London, London, United Kingdom

**Keywords:** biosynthesis of oxidized lipids, lipoxygenase (LOX), cyclooxygenase (COX), cytochrome P450, aldo-keto reductase (AKR), oxylipins, oxidized phospholipids, sterols and steroid hormones

## Abstract

Enzymatically oxidized lipids are a specific group of biomolecules that function as key signaling mediators and hormones, regulating various cellular and physiological processes from metabolism and cell death to inflammation and the immune response. They are broadly categorized as either polyunsaturated fatty acid (PUFA) containing (free acid oxygenated PUFA “oxylipins”, endocannabinoids, oxidized phospholipids) or cholesterol derivatives (oxysterols, steroid hormones, and bile acids). Their biosynthesis is accomplished by families of enzymes that include lipoxygenases (LOX), cyclooxygenases (COX), cytochrome P450s (CYP), and aldo-keto reductases (AKR). In contrast, non-enzymatically oxidized lipids are produced by uncontrolled oxidation and are broadly considered to be harmful. Here, we provide an overview of the biochemistry and enzymology of LOXs, COXs, CYPs, and AKRs in humans. Next, we present biosynthetic pathways for oxylipins, oxidized phospholipids, oxysterols, bile acids and steroid hormones. Last, we address gaps in knowledge and suggest directions for future work.

## Introduction

Lipids are a key component of life. They play essential roles in membrane structure, cell signaling and energy production. Like other biomolecules, they undergo chemical modifications that expand their functional repertoire. One modification that has been steadily gaining attention is lipid oxygenation, with interest being attributed to two factors: advances in analytical methods that allow detection of oxygenated species, and the ever-growing body of literature implicating them in biological processes and disease. “Enzymatically oxidized lipids” constitute a large portion of known oxygenated lipid mediators, being bioactive lipids that are produced locally through specific biosynthetic pathways in response to extracellular stimuli. Those derived from oxygenation of polyunsaturated fatty acids (PUFA) such as arachidonic acid (AA) include prostaglandins (PG), thromboxanes and leukotrienes, which function in modulating inflammation, the immune response, and hemostasis, and are broadly termed “oxylipins” (1, 2). Oxylipins also include multiply oxygenated derivatives of eicosapentaenoic acid (EPA) and docosahexaenoic acid (DHA), of which specific enantiomers have been termed “specialized pro-resolving mediators” (SPM) for their proposed roles in the resolution of inflammation (3). SPMs derived from DHA include D-resolvins, maresins and protectin. On the other hand, E-resolvins are proposed to be generated from EPA but the enzymatic pathways are unclear and require further investigation. Oxylipins also constitute a core functional group on larger lipids including oxidized phospholipids (oxPL), endocannabinoids and cholesteryl esters (CE). Oxidized CEs are conversely associated with atherosclerosis progression (4), whereas enzymatically oxidized phospholipids (eoxPL) are pro-coagulant and promote a variety of innate immune actions in leukocytes and platelets (5).

Enzymatically oxidized lipids are not limited to those containing oxygenated PUFA functional groups. Oxidized derivatives of cholesterol include steroid hormones, bile acids and their oxysterol precursors. Steroid hormones regulate multiple physiological processes including metabolism (e.g., glucose homeostasis by glucocorticoids), water retention, immune function, and development of sex characteristics (6–8). Bile acids, while traditionally known for aiding in fat digestion and bilirubin excretion, are also being increasingly revealed as signaling molecules and metabolic regulators (9, 10). Similarly, accumulating evidence is revealing oxysterols as more than just bile acid intermediates with functions in signaling (11).

Enzymatic lipid oxidation is facilitated by a network of proteins that use PUFAs or sterols as substrates, specifically, lipoxygenase (LOX), cyclooxygenase (COX), and cytochrome P450 (CYP), all of which exist as several isoforms exhibiting broad substrate specificity (12–16). In general, PUFA oxygenation is initiated by COXs, LOXs, and to a lesser extent CYPs. For example, the biosynthesis of PGs is initiated by COXs, whereas the formation of leukotrienes begins with a LOX. Additionally, crossover between COX, LOX, and CYP is proposed to produce various SPMs. On the other hand, cholesterol oxidation is dominated by CYPs. For example, sidechain shortening during steroid hormone synthesis is catalyzed by CYP11A1 (P450scc). Bile acids are synthesized from cholesterol predominantly by two pathways: the neutral pathway starting with an endoplasmic reticulum resident enzyme CYP7A1, and the acidic pathway starting with CYP27A1 in mitochondria (17). CYPs account for many enzymes in both pathways, catalyzing the formation of oxysterols as well as oxygenated PUFAs. Last, aldo-keto reductases (AKR) and hydroxysteroid dehydrogenases (HSD) catalyze key redox reactions in bile acid and steroid hormone biosynthesis.

By contrast, non-enzymatically oxidized lipids are produced through uncontrolled oxidation *via* free radical mechanisms. This involves the oxidation of lipids by free radicals, followed by chain propagation and ultimately termination. Notably, during oxygenation of PUFA, active site intermediates can escape to react in an uncontrolled manner, leading to some oxidized lipids forming due to non-enzymatic rearrangements of enzymatic pathway intermediates.

Here, we provide an overview of major enzymes involved in lipid metabolism, including LOXs, COXs, CYPs and AKRs. Then we outline the biosynthetic pathways of oxylipins, oxPLs, oxysterols, bile acids and steroid hormones. Finally, we address outstanding questions and suggest directions for future work.

## Enzyme Families Involved in the Biosynthesis of Oxidized Lipids

### Lipoxygenase (LOX)

#### Human LOX: Isoforms and Tissue Distribution

Lipoxygenases (LOX) are a family of non-heme iron-containing dioxygenases. They catalyze the stereospecific addition of dioxygen to lipids containing a (1Z,4Z)-pentadiene group producing lipid hydroperoxides. The human genome contains six functional LOX genes, expressed in various tissues (**Table 1**). LOXs were traditionally named according to their positional specificity for arachidonic acid (AA). However, the latest characterized member eLOX3 has limited lipoxygenase activity (38), while some LOXs show preference for other PUFA. Sequence analysis of human LOXs (using UniProt entries (39)) shows that 12R-LOX (*ALOX12B*) is evolutionarily closer to 15-LOX-2 (*ALOX15B*; 48.2% identity) than 12S-LOX (*ALOX12*; 35.7% identity). Additionally, human 15-LOX-1 (*ALOX15*) exhibits dual positional specificity which is not reflected in the name (18). The lack of a robust naming system is a common cause of confusion. Thus, the use of gene names alongside enzyme names is recommended (14).

**Table 1:** Human LOXs: Genes, substrates, and major expression sites.

Gene	Protein	Preferred substrate(s)	Expression sites	Refs.
*ALOX12*	12S-LOX	DHA & EPA > AA	Platelets, umbilical vein endothelial cells, vascular smooth muscle cells, skin epidermis	(18–22),
*ALOX12B*	12R-LOX	O-Linoleoyl-ω-hydroxyceramide AA & 8,11,14-eicosatrienoic acid > GLA	Hair roots, keratinocytes, B-cells, tonsil epithelial cells, bronchial epithelial cells	(23–27)
*ALOX15*	15-LOX-1 (12/15 LOX murine ortholog)	DHA > EPA > AA	Monocytes, macrophages, dendritic cells, eosinophils, reticulocytes, tracheal epithelium	(18, 28–32)
*ALOX15B*	15-LOX-2 (8-LOX murine ortholog)	DHA > EPA > AA	Macrophages, hair roots, prostate, lung, cornea, skin	(18, 28, 33),
*ALOX5*	5-LOX	AA & 5S-HpETE	Leukocytes, dendritic cells, mast cells, lung, placenta	(34–36)
*ALOXE3*	eLOX3	9R-Hydroperoxy-linoleoyl-ω-hydroxyceramide 12R-HpETE	Skin epidermis	(25, 37)

AA arachidonic acid DHA, docosahexaenoic acid; EPA, eicosapentaenoic acid; GLA, γ-linolenic acid; HpETE, hydroperoxy-eicosatetraenoic acid.

LOXs are typically constitutively expressed, except for 15-LOX-1 (*ALOX15*) which is inducible by IL-4 and IL-13 in monocyte-derived macrophages (28). Although 15-LOX-2 (*ALOX15B*) is constitutively expressed in the same cell type, its expression can be increased by cytokines, hypoxia, and lipopolysaccharide. It is possible that other constitutively expressed LOXs share this property.

#### Structure and Membrane Association of Mammalian LOXs

There are a limited number of published crystal structures for mammalian LOXs. Available structures of 5- and 15-LOXs show a single polypeptide chain that consists of two domains: a small β-barrel *N*-terminal domain and a larger α-helix-rich C-terminal domain containing the catalytic non-heme iron (40–42). The coordination positions of the catalytic iron are occupied by three conserved His residues, the carboxyl group of the C-terminus Ile residue, a water molecule and one last variable ligand (water, His, Asn, or Ser). Studies on mammalian LOXs found that the *N*-terminal domain is not required for catalytic activity, but instead functions in membrane binding and regulation (43, 44). The *N*-terminal domain of mammalian 5-LOXs (*ALOX5*) was found to be important for the calcium-dependent translocation from the cytosol or nucleus (depending on cell type) to the nuclear envelope and enzyme activity (45, 46). Similar findings were reported for mammalian 15-LOXs, with the translocation from the cytosol to the cell membrane instead (47, 48). Unlike other LOXs, the activity of 5-LOX requires interaction with partner proteins 5-lipoxygenase activating protein (FLAP), coactosin-like protein (CLP) and cytosolic phospholipase A2 (cPLA_2_) (49, 50). Additionally, 5-LOX undergoes phosphorylation at multiple sites, regulating its translocation and activity, and is allosterically activated by ATP (51–53).

On the other hand, 15-LOXs do not require accessory proteins for free FA oxygenation. Instead, 15-LOXs form a complex with a small scaffolding protein, phosphatidylethanolamine-binding protein 1 (PEPB1) (54). This complex facilitates 15-LOX activity on PL-esterified PUFA and is proposed to play regulatory roles in ferroptosis along with GPX. PEPB1 has been suggested to direct 15-LOX activity toward PL substrates when free AA is depleted (55). The crystal structure of human 15-LOX-2 (*ALOX15B*) revealed a hydrophobic loop in the *N*-terminal domain that is flanked by calcium binding sites, a feature that is absent in 5-LOX (42). Hydrogen-deuterium exchange mass spectrometry showed a decrease in H/D exchange for the hydrophobic loop, supporting a role in membrane hopping (48, 56). Both 5-LOX and 15-LOXs associate with membranes in a calcium-dependent process but they exhibit differences in activity, translocation and binding partners.

Calcium-dependent membrane association has also been shown for platelet 12S-LOX (*ALOX12*), and more recently, 12R-LOX (*ALOX12B*), and eLOX3 (19, 57). The activity of 12S-LOX (*ALOX12*) is thought to be regulated by the availability of its substrates, which are supplied through the action of phospholipases. It is currently unknown whether 12S-LOX is regulated by other mechanisms. Research on the regulation of 12R-LOX and eLOX3 is also limited, although calcium has been shown to increase the activity of mouse 12R-LOX but not eLOX3 (58).

Aside from the catalytic domain of 12S-LOX (*ALOX12*), no crystal structures are available for 12-LOXs or eLOX3. However, human platelet 12S-LOX (*ALOX12*) was characterized using small-angle x-ray scattering, showing its occurrence as a dimer in solution (59). This challenged the idea that all human LOXs exist and function as monomers. Human 5-LOX displays full activity as a monomer but forms functional dimers as well, thought to exist in equilibrium (60). Similarly, rabbit 15-LOX-1 (*ALOX15*) undergoes ligand-induced dimerization in aqueous solutions, and molecular dynamics predicts stable dimers in the presence of substrate fatty acids (61). This supports the idea of monomer-dimer equilibria in LOXs. Human 15-LOX-1 (81.1% sequence identity to rabbit) likely exhibits the same property.

In summary, all six human LOXs associate with membranes in a calcium-dependent manner. 5-LOX requires the help of protein partners for membrane translocation and activity, whereas 15-LOXs only require a partner protein to efficiently catalyze the oxygenation of PL-esterified PUFA. Less is known about the regulation of 12-LOXs and eLOX3. Dimerization and monomer-dimer equilibria have been observed in several LOX isoforms and represent a potential regulatory mechanism. Finally, complete crystal structures have yet to be reported for 12S-LOX, 12R-LOX, and eLOX3. In the case of 12S-LOX, a crystal structure would be beneficial in the rational design of inhibitors, as the enzyme functions in platelet activation.

#### Reaction Mechanism of LOX

**Figure 1:**
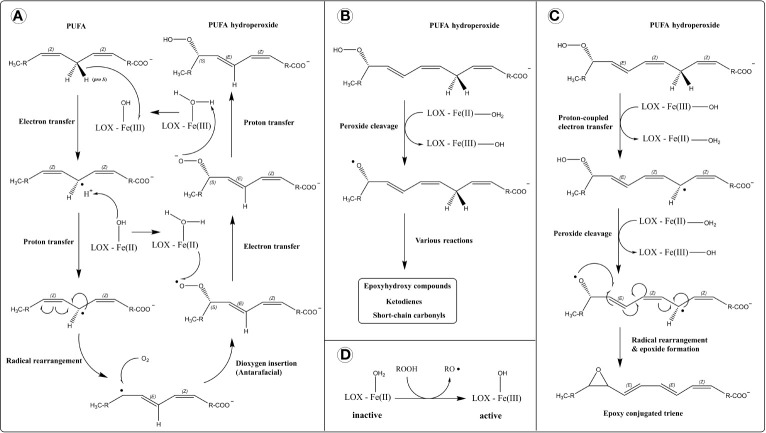
Reaction mechanism of lipoxygenases (LOXs). **(A)** Dioxygenase activity. Hydrogen atom removal is thought to proceed through a proton-coupled electron transfer mechanism (PCET), in which the transfer of electron and proton occur simultaneously (concerted mechanism), depicted separately for simplicity. **(B)** Lipohydroperoxidase activity. **(C)** Leukotriene synthase activity. **(D)** Activation of LOX by a hydroperoxide.

The dioxygenase activity of LOX represents a highly controlled form of lipid peroxidation (**Figure 1A**). First, stereospecific hydrogen atom removal is carried out by the non-heme ferric iron [Fe(III)]. Fe(III) is not a strong enough oxidizer to abstract hydrogen directly. Thus, a mechanism involving proton-coupled electron transfer (PCET) has been proposed (62) (**Figure 1A**). In this, the electron is directly transferred to Fe(III) and the proton is picked up by the hydroxide ligand in a concerted mechanism (simultaneously), producing a lipid alkyl radical and ferrous iron [Fe(II)]. This is followed by a rearrangement of the lipid radical into the more stable conjugated diene. After that, dioxygen is introduced onto the opposite side of the removed hydrogen (antarafacially), generating a lipid peroxyl radical. Finally, the lipid peroxyl radical is reduced by Fe(II) and protonated to form a lipid hydroperoxide, through PCET. This reforms Fe(III) for another round of catalysis.

The stereo and regio-specificity of lipoxygenation are determined by structural features which: (i) accommodate and position the substrate in a specific orientation, and (ii) direct radical rearrangement and oxygen insertion. These features include a U-shaped cavity that accommodates the substrate (63), a migration channel for oxygen (64), a glycine/alanine “switch” that determines stereospecificity by directing oxygen (65), and several amino acid residues that control positional specificity (66, 67). These properties are explored in-depth by Newcomer and Brash (68).

In addition to classical dioxygenase activity, LOXs catalyze other reactions that involve free-radical processes like hydrogen abstraction, homolytic bond cleavage, and radical rearrangement (69). eLOX3 exhibits a hydroperoxide isomerase activity (lipohydroperoxidase activity) which converts hydroperoxides to epoxy-alcohols and ketones (70) (**Figure 1B**). eLOX3 has restricted dioxygenase activity (38), and its hydroperoxide isomerase activity is considered its primary catalytic reaction. Also, mammalian 5-LOXs and human 15-LOX-1 (*ALOX15*) possess leukotriene A_4_ synthase activity which produces epoxides from hydroperoxides (71–74). The mechanism involves the homolytic cleavage of a hydroperoxide into an alkoxy radical and hydrogen atom removal (*via* PCET) from a bis-allylic methylene carbon generating an alkyl radical. The resulting biradical is stabilized by epoxide formation (**Figure 1C**).

LOXs are inactive in their basal form with Fe(II) and require activation *via* oxidation into Fe(III) (**Figure 1D**). LOX activation is facilitated by hydroperoxides such as their dioxygenase reaction products. Although the proposed LOX catalytic cycle regenerates the ferric iron for another round of catalysis, small amounts of radical intermediates can escape the active site resulting in an incomplete catalytic cycle and requiring repeated activation of the enzyme (formation of Fe(III) by hydroperoxide) for sustained catalysis (69, 75, 76).

Some LOXs exhibit suicide inactivation, a property in which the reaction product rapidly inactivates the enzyme. Human 5-LOX is irreversibly inactivated by both of its products 5-HpETE and leukotriene A_4_ (77). Similarly, rabbit reticulocyte 15-LOX (human 15-LOX-1 ortholog) is inactivated by its product 15-HpETE, and the mechanism is suggested to be through formation of reactive intermediates that covalently bind the enzyme (78). The dioxygenase reaction and the other two activities (lipohydroperoxidase and leukotriene synthase) have been proposed to inactive LOXs through different mechanisms, but the mechanistic details remain unclear (78).

#### LOXs Act on a Broad Range of Substrates

LOX isoforms have different substrate preferences. Substrates of mammalian 5-LOXs include AA, its hydroperoxide product 5*S*-HpETE, as well as epoxy-alcohols derived from EPA and DHA (71, 79). The former two are important in the biosynthesis of leukotrienes, while the latter two are proposed to be precursors of E- and D-series resolvins, respectively (75, 80). 15-LOXs (*ALOX15* and *ALOX15B*) accept AA, EPA, DHA, linoleic acid (LA), and γ-linolenic acid (GLA) as substrates. The preference of human 15-LOX orthologs is: DHA > EPA > AA > GLA > LA (18). One key difference is that 15-LOX-1 (*ALOX15*) possesses dual positional specificity for some of its substrates (e.g., 12 and 15-lipoxygenase activities for AA), whereas 15-LOX-2 (*ALOX15B*) exhibits singular specificity (15-lipoxygenase activity) (55). The hydroperoxide isomerase and epoxidase activities of 15-LOX-1 (*ALOX15*) are proposed to function in the synthesis of eoxins, E-series resolvins, and protectin from their respective precursors. Additionally, human 15-LOX-1 oxygenates PUFA-containing fatty amides and 2-arachidonoyl-glycerol ester (81, 82). Finally, mammalian 15-LOXs can catalyze the oxygenation of PUFA in lysophospholipids (lysoPL), PLs, CEs, and lipoproteins (83–88).

PUFA substrates of platelet 12S-LOX (*ALOX12*) include AA, EPA, DHA, and dihomo-γ-linolenic acid (DGLA) (18, 89). While no single study has compared all substrates at the same time, one determined the order of preference for the first three substrates to be: DHA > EPA > AA (18). Another found comparable kinetic parameters for EPA, AA, and DGLA as substrates (89). Both observed a singular positional specificity for 12S-LOX. LA, GLA, and α-linolenic acid (ALA) were all found to be poor substrates for 12S-LOX (18, 89, 90). Furthermore, human 12S-LOX possesses lipoxin synthase activity, which converts leukotriene A_4_ into lipoxins A and B (91). The enzymatic activities of 12S-LOX are also proposed to function in the biosynthesis of hepoxilins and maresins (92, 93). Recently, 12S-LOX was shown to oxygenate 2-AA-lysoPL, proposing alternate pathways for oxylipin and oxPL biosynthesis (94).

Mammalian 12R-LOX (*ALOX12B*) and eLOX3 (*ALOXE3*) are co-expressed in skin and their functions are related. 12R-LOX efficiently catalyzes the peroxidation of AA, DGLA and GLA, and less efficiently LA, EPA and DHA (23, 24). Later, 12R-LOX was shown to oxygenate LA esterified to ω-hydroxyacyl-sphingosine more efficiently than free LA (25). The hydroperoxide product of this reaction is further converted into an epoxy-alcohol by the isomerase activity of eLOX3. These two reactions are essential for the proper formation of the water-impermeable barrier in corneocytes (25). Human eLOX3 isomerase activity has also been observed on the 12*R*- and 12*S*-hydroperoxides of AA, with the reaction occurring 2-3 times faster for the *R*-stereoisomer (95). These reactions produce hepoxilins, which play a role in skin inflammation. Curiously, mouse 12R-LOX and eLOX3 metabolize the methyl esters of AA and LA more efficiently than the unmodified FAs (58). However, human and mouse orthologs are known to exhibit differences in substrate preference and regio-specificity (95). Thus, further work is needed to assess the ability of human 12R-LOX to metabolize fatty methyl esters and whether that reaction is biologically relevant.

Several aspects of LOX enzymology remain unresolved. First, the functional implications of suicide inactivation are unknown, that is, the reason why some LOXs have not evolved to resist suicide inactivation. This suggests a biological function for this property, perhaps in regulation. Additionally, the mechanistic details of suicide inactivation are not fully understood. Second, due to the ever-increasing list of LOX substrates, it has been difficult to pinpoint biologically relevant substrates and assign clear functions to all LOX isoforms. For example, the function of 15-LOX-2 (*ALOX15B*) in macrophages remains unclear, although it has been recently shown to regulate cholesterol levels (96). Unique substrates of each isoform (e.g., CEs for 15-LOXs, lysoPLs for 12S-LOX) might be worth investigating.

### Cyclooxygenase (COX)

#### Human COX: Isoforms and Tissue Distribution

Cyclooxygenases (COXs), also called prostaglandin-endoperoxide synthases and prostaglandin G/H synthases, are heme-containing enzymes that possess both oxygenase and peroxidase activities. There are two COX genes in humans: *PTGS1* and *PTGS2*, which encode for COX-1 and COX-2, respectively. Traditionally, it was thought that COX-1 is constitutively expressed, whereas COX-2 is inducible in response to inflammatory signals. However, several studies suggest constitutive COX-2 expression in the brain, lungs, gut, thymus, kidneys, and blood vessels (97–99). In the vasculature, COX-2 has been demonstrated to be a key source of vascular prostacyclin (100, 101). COX-1 is ubiquitously expressed in the body, and its expression sites include blood vessels, prostate, immune cells (monocytes, T-cells), platelets, stomach, resident inflammatory cells, smooth muscles, and mesothelium of many organs (102–105). On the other hand, COX-2 is inducible in many tissues including prostate, immune cells (T-cells, B-cells, monocytes), and stomach (102–104, 106), but also constitutively expressed in some tissues as previously mentioned.

#### Structure of COX

The role of COXs as mediators of inflammation and their potential as drug targets were drivers for the elucidation of their structures. The crystal structure of sheep COX-1 has been solved in the presence and absence of various synthetic ligands (107–109). Similarly, there are available structures for mouse COX-2 in the presence and absence of natural and synthetic ligands (13, 110–113), as well as human COX-2 with several inhibitors (114, 115).

Both COX isoforms are homodimers, and this quaternary structure is necessary for enzymatic activity (116). Each monomer consists of three domains: An *N*-terminal epidermal growth factor (EGF)-like domain, a membrane binding domain, and a large C-terminal catalytic domain. The EGF-like domain is located at the dimer interface and potentially facilitates dimerization. It is also thought to facilitate membrane binding (117). The membrane binding domain consists of four amphipathic α-helices that insert into one face of a membrane bilayer. Both COXs are found on the luminal face of the endoplasmic reticulum (ER) and the inner and outer nuclear membranes (118). However, COX-2 is also found in the Golgi apparatus (119). The catalytic domain (in both isoforms) contains separate oxygenase and peroxidase active sites on opposite sides of the heme cofactor. The oxygenase active site is located towards the membrane binding face at the end of a hydrophobic tunnel that allows substrate entry, whereas the peroxidase active site is in a groove on the opposite face of the enzyme (120).

COXs undergo *N*-glycosylation at multiple asparagine residues. This facilitates their proper folding, and regulates the turnover of COX-2 by controlling its trafficking between the ER and the Golgi apparatus (119, 121, 122). COX-2 can also be *S*-nitrosylated by inducible nitric oxide synthase, which has been proposed to enhance its activity (123). *In vitro* experiments on recombinant human COX-1 and COX-2 demonstrated that both isoforms can be *S-*nitrosylated by a nitric oxide donor, but only COX-2 showed increased cyclooxygenase activity (124). Circular dichroism data suggest an altered, less dynamic conformation for both isoforms after *S-*nitrosylation with less α-helices, turns, and random coils, but more β-sheets. The structural change was more pronounced in COX-2 compared to COX-1. These findings provide new insight into the structural dynamics of COXs. The occurrence and biological relevance of this altered COX conformation is unexplored *in vivo* and would benefit from further investigation.

Although COXs are sequence homodimers, they are considered conformational heterodimers because one monomer functions as the catalytic subunit (E_cat_) and the other as an allosteric regulator (E_allo_) (125–127). For both COX isoforms, the binding of one heme molecule to E_cat_ is required for full activity. Several substrate and non-substrate FAs can bind E_allo_ to regulate the activity of E_cat_, and the two COX isoforms exhibit differences in this regard. COX-1 is inhibited by palmitic acid, stearic acid, margaric acid and oleic acid (127), none of which are COX substrates. On the other hand, COX-2 is stimulated by palmitic acid and stearic acid (126). Substrate FAs (like AA and EPA) also bind to E_allo_ to regulate COX activity, with notable differences in the responses of the two isoforms (reviewed in (128)).

#### Catalytic Mechanism of COX

**Figure 2:**
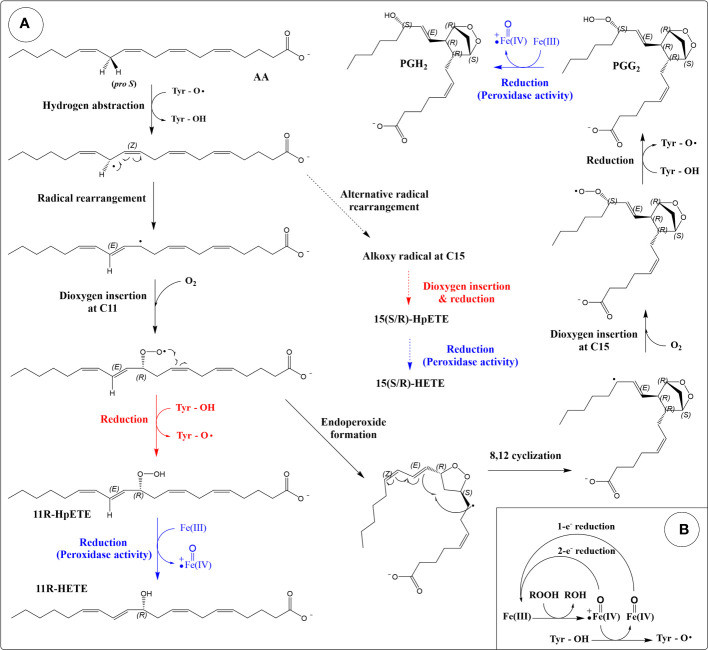
Reaction mechanism of cyclooxygenases (COXs). **(A)** Production of eicosanoids from arachidonic acid through the dioxygenase and peroxidase activities of COX. The cyclooxygenase reaction is colored black. Peroxidase reactions are coloured blue. Reactions that produce HpETEs are colored red. Side reactions that produce 15-HETEs are depicted by dashed arrows. **(B)** The peroxidase cycle generates the Tyr radical required for hydrogen abstraction (porphyrin ring of heme not shown).

COXs possess two enzymatic activities: a dioxygenase activity and a peroxidase activity. The dioxygenase activity is a controlled peroxidation process (129), and the role of the enzyme is to direct hydrogen abstraction and the stereochemistry of formation of intermediates (120). Unlike LOXs where hydrogen abstraction is carried out by a non-heme associated Fe(III), hydrogen abstraction in COX is carried out by a catalytic tyrosyl radical (**Figure 2A**). The heme group oxidizes a tyrosine residue in the active site into a radical, which then abstracts a hydrogen (stereo- and regio-specifically) from the (1Z,4Z)-pentadiene group of the lipid. Using AA as an example substrate, the 13-*pro*-S hydrogen is abstracted. The resulting alkyl radical rearranges into a conjugated diene before the addition of dioxygen at C11, generating a peroxyl radical (*11R*-stereochemistry). Rotation of the peroxyl radical positions the outer oxygen atom in the correct orientation to attack the C9 carbon resulting in an endoperoxide (130). A second cyclization involving C8 and C12 generates a bicyclic ring and a radical at C15. Note that the cyclopentane ring is formed in the trans configuration (131). This differs from the non-enzymatically generated isoprostanes which occur predominantly in the cis configuration (132). Following the second cyclization, dioxygen is added onto the *si* face of the C15, forming a peroxyl radical (*15S*-stereochemistry). Hydrogen atom transfer from the active site tyrosine residue forms prostaglandin G_2_ (PGG_2_) and regenerates the tyrosyl radical for another round of catalysis.

The peroxidase activity of COX reduces PGG_2_ into PGH_2_ (**Figure 2A**). The mechanism involves a two-electron reduction of the hydroperoxide into an alcohol, with a corresponding oxidation of the heme group into the oxoferryl form (133) (**Figure 2B**). Regeneration of the heme group can be achieved by two sequential one-electron reductions. The peroxidase activity is required for the dioxygenase activity as the oxidation of the tyrosine into a radical is done by the oxoferryl-porphyrin cation radical generated during the peroxidase reaction cycle (133) (**Figure 2B**). Suicide inactivation has been described for both the dioxygenase and peroxidase activities of COX (133, 134).

The dioxygenase activity of COX can also result in a lipoxygenase-type reaction, in which one dioxygen molecule is introduced and no endoperoxide formation occurs (135, 136) (**Figure 2A**). In this case, the peroxyl radical formed after the addition of the oxygen is reduced into a hydroperoxide instead of participating in the cyclization reaction. This reaction represents an incomplete catalytic cycle and occurs as a side product. Using AA as a substrate, this lipoxygenase-type reaction (after reduction by peroxidase) leads to the formation of 11*R*-HETE (HETE: hydroxy-eicosatetraenoic acid), 15*R*-HETE and 15*S*-HETE (minor product compared to 15*R*-HETE in both isoforms) (137). C11 and C15 are the same oxygen insertion sites in the dioxygenase (cyclooxygenase reaction), and these by-products are thought to arise from alternate conformations of substrate in the active site (138).

Aspirin (acetylsalicylic acid) is an inhibitor of COXs, and its mechanism of action involves the acetylation of a serine residue (Ser530 in COX-2) in the active site. Acetylation of COX-1 leads to complete inhibition (139), whereas the acetylation of COX-2 promotes the lipoxygenase-type reaction and the formation of 15*R*-HETE (140). It was previously thought that acetylation completely inhibits PG production by COX-2 (cyclooxygenase reaction). However, recent evidence shows that the acetylated enzyme retains that activity (15*S*-PG formation) but also more favorably forms 15*R*-PGs (141), although PGs are still minor products compared to 15*R*-HETE for acetylated-COX-2. These findings are consistent with an earlier study that reported the formation of 15*R*-PGE_2_ in COX-2 Ser530 mutants (137). Thus, both acetylation and mutagenesis of Ser530 in COX-2 promote 15*R*-PG formation.

#### COX Isoforms Differ in Their Substrate Specificity

COXs catalyze the transformation of AA into PGH_2_, which is a precursor of other PGs (PGD_2,_ PGE_2_, PGF_2α_, PGI_2_) and thromboxane. These oxygenated AA derivatives are generated through the action of tissue-specific enzymes downstream of COX (discussed in section 3.1) and play roles in inflammation, blood flow regulation and blood clotting through interactions with specific GPCRs (142).

COXs also accept other PUFAs as substrates including DGLA, LA, ALA, EPA, and GLA (143), at least *in vitro*. An early study determined the efficiency of substrate utilization for human COXs (K_cat_/K_m_) to be AA > DGLA > LA > ALA, with ALA being a poor substrate for COX-1. EPA and GLA are poor substrates for both isoforms but they are better substrates for COX-2 than for COX-1 (143). Later studies have tested other PUFAs like eicosadienoic acid, adrenic acid, docosapentaenoic acid and DHA as COX substrates (127, 144). In general, COX-2 was found to be more efficient than COX-1 for a broader range of PUFAs. That said, AA is the preferred substrate for both isoforms, but COX-2 can oxygenate it at lower concentrations compared to COX-1 due to differences in their allosteric regulation by FAs (145).

COX-2 can also catalyze the 11*R*-, 15*R*-, 15*S*-dioxygenation and bis-oxygenation of 5*S*-HETE, forming diHETEs in the former three reactions and a di-endoperoxide product in the later reaction. However, acetylation of COX-2 shifts the specificity into favoring 15*R*-dioxygenation producing 5*S*,15*R*-diHETE (146). Additionally, COX-2 can oxygenate AA in complex lipids for example; arachidonoylethanolamide, 2-arachidonoylglycerol (2-AG), and N-arachidonoyl-glycine (147–149). Recently, 2-AA-lysoPL, and ethanolamide derivatives of EPA and DHA were also shown to be COX-2 substrates (88, 150). Oxygenation of AA-lysoPL by COX-2 generates eicosanoid-lysoPL, which can be hydrolyzed to release eicosanoids through intracellular lipases (88). The ability of COX-2 to bind and oxygenate a broader range of substrates compared to COX-1 has been attributed to a larger active site, the orientation of an Arg residue in the substrate binding pocket and amino acid residues lining the tunnel leading to the cyclooxygenase active site (13).

COX isoforms exhibit differences in expression, tissue distribution, allosteric regulation, and substrate specificity. However, some aspects of COX biochemistry are unclear. For example, the physiological functions of COX-2-derived oxygenated endocannabinoids are unclear. Additionally, COX-2 oxygenates AA-lysoPLs into eicosanoid-lysoPLs, which are proposed to function in signaling and as precursors to other mediators (88). However, the metabolism of eicosanoid-lysoPLs requires further investigation.

### Cytochrome P450 (CYP)

#### Human CYPs: Nomenclature and Tissue Distribution

Cytochrome P450s (CYP) are a superfamily of heme-containing monooxygenases that are ubiquitous across all domains of life. There are 57 functional CYP genes in humans, and their products are further divided into 18 families and 41 sub-families based on amino acid sequence (151, 152). A robust, unified nomenclature system has been devised for CYPs, encompassing all known CYPs across living organisms (153, 154). The name includes the root “CYP”, followed by a number for family, a letter for subfamily, and a gene-identifying number for isoforms. Additionally, an asterisk and a number are added at the end to denote alleles (155). Families and subfamilies are based on 40% and 55% amino acid sequence identity, respectively.

CYPs are widely distributed in mammalian tissues (156–158), with a particularly high expression in the liver, brain, kidney and lung. Intracellularly, mammalian CYPs are generally bound to mitochondrial membranes and the endoplasmic reticulum (ER; microsomes when *in vitro*) (159, 160). There is also evidence of mammalian CYPs in other compartments including the plasma membrane and the nucleus, based on *in vitro* studies on cultured cells (161–164). Some microsomal CYPs are also targeted into the mitochondria (159). In the human genome, 50 out of the 57 functional CYP genes code for microsomal CYPs, and the remaining seven for mitochondrial isoforms. Some CYPs are inducible by environmental stimuli, whereas others are constitutively expressed (152). Induction of CYP expression by environmental compounds can be through interactions with nuclear receptors, transcriptional regulatory elements, or non-coding RNAs (165).

Two major functions of CYPs are in drug metabolism and lipid metabolism. Here, we focus on lipid metabolism which involves members of most human CYP families. For an extensive review, the reader is referred to Nelson and Nebert (166).

#### Structure of Mammalian CYPs

Structural characterization was first carried out on bacterial CYPs, which are typically water soluble. On the other hand, mammalian CYPs are membrane-bound, and initial attempts to crystallize them were unsuccessful until a small *N*-terminal hydrophobic helical segment was replaced with a hydrophilic sequence from a related protein. The first structure to be published was of rabbit CYP2C5, a microsomal CYP isoform that hydroxylates progesterone (167). Comparison of CYP2C5 structure with closely related microbial CYPs showed a similar folding geometry, but unique features of the mammalian enzyme were observed such as a hydrophobic surface that was proposed to play a role in membrane binding (167).

CYPs consist of two domains: a β-sheet-rich *N*-terminal domain and a larger helix-rich C-terminal catalytic domain. The *N*-terminal domain in microsomal CYPs contains a transmembrane helix that plays a role in membrane anchoring, but that role is not exclusive as CYPs contain other regions (in the catalytic domain) that bind the membrane. This transmembrane helix is not present in mitochondrial CYPs, and their membrane binding is facilitated by hydrophobic and amphipathic regions on the surface (160, 168). The *N*-terminus also contains a signal peptide sequence for trafficking into the appropriate compartment, that in the case of mitochondrial CYPs, is cleaved during transport. The catalytic domain contains a deep cavity that houses the heme prosthetic group. A thiolate ion in a conserved cysteine residue occupies the fifth coordination position of heme, and a water molecule occupies the sixth position in the resting state. There is variable flexibility in the active site among CYPs, and a high degree of flexibility has been correlated to substrate promiscuity (169, 170). Residues in helices surrounding the active site heme position the substrate for catalysis. Additionally, in the CYP4 family, a covalent linkage has been identified between the heme group and a conserved glutamic acid residue (171), and this interaction plays a role in substrate positioning for ω-hydroxylation (hydroxylation at the methyl end of FAs) (172).

Other structural features of CYPs include access channels that allow entry of substrates from both the aqueous and membrane compartments and an exit channel for the product (160). Furthermore, oligomerization is well-documented in both microsomal and mitochondrial CYPs, which can be between subunits of the same CYP (homomeric; e.g., CYP2C2) or different CYPs (heteromeric; e.g., CYP2E1/CYP3A4 and CYP1A2/CYP2B4) (173–180). These interactions provide an additional layer of regulation and play a role in substrate specificity.

#### Enzymology of Mammalian CYPs

CYPs catalyze a wide range of reactions (181, 182), but the most common include C-hydroxylation, heteroatom oxidation, heteroatom dealkylation, epoxidation, and group migration (182). Many reactions of CYPs are dependent on their ability (owing to the thiolate-heme group) to catalyze the scission of dioxygen and incorporate one of the oxygen atoms into the substrate, with the other being reduced to water. These reactions require electron transfer from a donor, NADPH, to the heme iron. However, transfer of electrons from NADPH to CYPs requires protein partners: NADPH cytochrome P450 reductase in the case of microsomal CYPs, and the combined functions of adrenodoxin reductase and adrenodoxin in mitochondrial CYPs. Thus, CYPs form complexes with their redox partners, at least during catalysis. That said, not all CYP reactions require external oxygen or electron donors. Examples are isomerization reactions catalyzed by CYP5A1 (thromboxane synthase) and CYP8A1 (prostacyclin synthase) (183), two enzymes involved in PGH_2_ metabolism.

**Figure 3:**
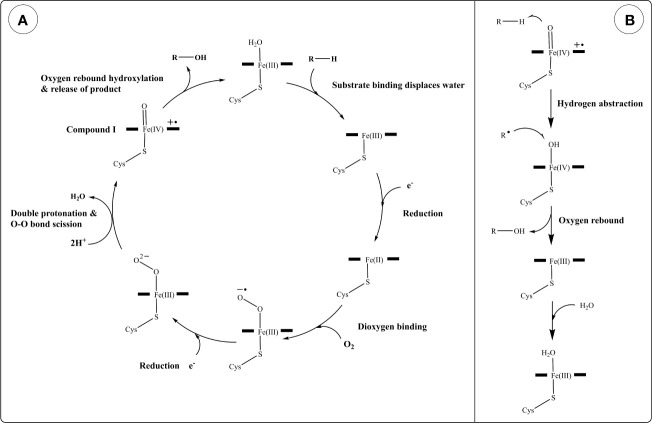
Reaction mechanism of cytochrome P450s (CYPs). **(A)** Generalized catalytic cycle of CYPs. **(B)** Hydroxylation of a generic substrate (R–H) by compound I *via* an oxygen rebound mechanism.

A generalized reaction mechanism for CYPs (**Figure 3A**) contains the following steps: starting with the resting state, substrate recruitment displaces H_2_O from the 6^th^ (axial) position of the heme Fe(III). This is followed by a 1-electron reduction from a donor forming Fe(II), O_2_ binding, and another 1-electron reduction resulting in a negatively charged peroxo group. Protonation of this complex twice by nearby H_2_O or amino acid residues results in the scission of the dioxygen (O-O bond) into an oxoferryl intermediate (compound I) and a water molecule (184). Compound I is thought to be the direct oxidant in many CYP oxidation reactions (185). In hydroxylation reactions, compound I abstracts a hydrogen from an alkyl group in the substrate generating a carbon radical and an iron oxygen complex, which rapidly react together forming a hydroxyl group on the substrate and regenerating the heme iron into the Fe(III) state (**Figure 3B**). Although this is a generalized reaction scheme for CYPs, the intermediates following O_2_ binding until compound I have been difficult to characterize due to their instability and the exact electronic structures are not fully assigned (185).

#### Mammalian CYPs Play Crucial Roles in Oxidative Lipid Metabolism

CYPs are involved in the metabolism of a wide range of lipids including PUFAs and sterols (**Table 2**). Hydroxylation and epoxidation are two common CYP-mediated reactions for PUFA substrates. CYPs can hydroxylate FAs terminally (ω-hydroxylation) or midchain. Similarly, CYP-mediated epoxidation can occur on various double bonds in PUFA. CYPs exhibit variable preference with respect to the reaction type and positional specificity. CYP1-3 families catalyze epoxidation and hydroxylation reactions for FAs, whereas the CYP4 family favors hydroxylation over epoxidation, specifically ω-hydroxylation (204).

**Table 2:** Examples of human cytochrome P450s (CYPs) and their involvement in lipid metabolism.

Enzyme	Lipid substrate(s)	Reaction(s)	Refs.
CYP1A1	AA, EPA, DHA	Epoxidation & hydroxylation	(186)
CYP2C8	AA, EPA, DPA, DHA	Epoxidation & hydroxylation	(186–188)
CYP3A4	Sterols	Hydroxylation	(189–191)
CYP4A11	Lauric acid, PA AA, EPA, DPA, DHA	ω-Hydroxylation ω/ω-1-Hydroxylation	(188, 192),
CYP4F2	AA, EPA, DPA, DHA	ω/ω-1-Hydroxylation	(188)
CYP5A1 (thromboxane synthase)	PGH_2_	Isomerization	(183)
CYP7A1	Cholesterol, 7-DHC	7α-Hydroxylation, epoxidation, carbonylation	(161, 193, 194)
CYP7B1	Oxysterols, steroids	7α-Hydroxylation	(195)
CYP8A1 (prostacyclin synthase)	PGH_2_	Isomerization	(183)
CYP8B1	Sterols	12α-Hydroxylation	(196)
CYP11A1 (P450scc)	Cholesterol	Side-chain cleavage	(197)
CYP11B1	11-Deoxycortisol, 11-deoxycorticosterone	11β-Hydroxylation	(198)
CYP11B2	11-Deoxycorticosterone	11β-Hydroxylation, 18-hydroxylation & oxidation	(198)
CYP17A1	Pregnenolone, progesterone	17-Hydroxylation	(199)
CYP19A1 (P450arom/aromatase)	Testosterone	Aromatization	(199)
CYP21A2	Progesterone	Hydroxylation	(199)
CYP24A1	Calcitriol	24-Hydroxylation	(200)
CYP26A1	all-*trans*-Retinoic acid	Hydroxylation	(201)
CYP27A1	Sterols	(25R)26-Hydroxylation and carboxylation	(197)
CYP39A1	24-hydroxycholesterol	7α-Hydroxylation	(17)
CYP46A1	Cholesterol, desmosterol	24S-Hydroxylation, 24S-epoxidation	(197, 202)
CYP51A1	Lanosterol	14α-Demethylation	(203)

7-DHC, 7-dehydrocholesterol; AA, arachidonic acid; EPA, eicosapentaenoic acid; DPA, docosapentaenoic acid; DHA, docosahexaenoic acid; PA, palmitic acid; PGH_2_, prostaglandin H_2_.

Terminal hydroxylation of AA by CYPs produces 20-hydroxyeicosatetraenoic acid (20-HETE), which functions in blood pressure regulation and water balance (205). Additionally, terminal hydroxylation functions in ω-oxidation (a catabolic pathway for FAs), the degradation of some eicosanoids (206, 207), and the proper formation of the skin permeability barrier (208). Midchain hydroxylation of PUFA produces mono-hydroxylated derivatives (HETEs in the case of AA) with various bioactivities (reviewed in (152)). Unlike LOXs and COXs which oxygenate carbons two bonds away from bis-allylic methylene carbons, CYPs can also oxygenate bis-allylic carbons (producing 7-, 10- and 13-HETE from AA), and various other positions (209–211). However, bis-allylic hydroxylation products are unstable in mildly acidic conditions and rearrange into conjugated dienes (211).

Epoxidation of AA by CYPs produce epoxy-eicosatrienoic acids (EET). However, other PUFA like LA, EPA and DHA also undergo CYP-mediated epoxidation. Epoxy derivatives of PUFAs are implicated in blood pressure regulation and inflammation (15). Their metabolism is undertaken by several epoxide hydrolases including soluble epoxide hydrolase (sEH) and microsomal epoxide hydrolase (mEH) (212). The action of epoxide hydrolases produces dihydroxy derivatives which possess different bioactivities from their epoxide precursors (15).

Arachidonoylethanolamide also undergoes CYP hydroxylation and epoxidation (213, 214). Similarly, CYPs catalyze epoxidation of EPA and DHA ethanolamides, producing compounds with anti-inflammatory and anti-angiogenic effects (215). These lipids can be degraded by both sEH and fatty acid amide hydrolase (FAAH) (215), suggesting a link between the CYP epoxygenase and endocannabinoid pathways. The metabolism and biological roles of these mediators is an active area of research (216).

CYPs catalyze several types of reactions in the metabolism of sterols, some of which involve multiple oxygenation/hydroxylation steps. Demethylation of lanosterol by CYP51A1 is a key reaction in cholesterol biosynthesis and involves three successive oxidations of a methyl group, resulting in its release as formic acid and the introduction of a double bond at the D ring (217). Similarly, the formation of estradiol from testosterone *via* the action of CYP19A1 (P450arom) involves the elimination of a methyl group by three successive oxidation steps and the introduction of a double bond at the A ring. Additionally, cholesterol side-chain cleavage by CYP11A1 (P450scc) involves two hydroxylation steps on different carbons followed by a C-C bond cleavage (218). This reaction produces pregnenolone which is a key intermediate in the formation of androgens, estrogens, glucocorticoids and mineralocorticoids.

CYP-mediated hydroxylation reactions are ubiquitous in cholesterol metabolism, leading to the formation of oxysterols, steroid hormones and bile acids. Key enzymes in the bile acid biosynthesis pathways are CYP7A1 (cholesterol 7α-hydroxylase), CYP8B1 (sterol 12α-hydroxylase), CYP27A1 (commonly named sterol 27-hydroxylase, but more correctly sterol (25R)26-hydroxylase) and CYP7B1 (oxysterol 7α-hydroxylase) (219, 220). Of these enzymes, CYP27A1 can both hydroxylate and carboxylate the terminal carbon of the sterol side-chain (221). Other enzymes involved in quantitatively minor bile acid biosynthesis pathways are CYP46A1 (cholesterol 24S-hydroxylase) which 24S-hydroxylates cholesterol (222, 223) and CYP3A4 which has 4β- and 25-hydroxylase activity (189, 224). CYP enzymes involved in steroid hormone biosynthesis include CYP11A (P450scc) (225), CYP17A1 (steroid 17α-hydroxylase) (226), CYP21A2 (steroid 21-hydroxylase), CYP11B1 (steroid 11β-hydroxylase), and 11B2 (aldosterone synthase) and CYP19A1 (aromatase) (198).

Thus, CYPs play indispensable roles in the metabolism of both PUFA and cholesterol. However, some human CYPs like CYP2A7 and CYP20A1 have no assigned functions and remain orphan enzymes.

### Human Aldo-Keto Reductases (AKRs) and Hydroxysteroid Dehydrogenases (HSDs)

#### AKRs: Nomenclature, Genes, and Tissue Distribution

Aldo-keto reductases (AKRs) are a superfamily of NADPH-dependent oxidoreductases. They catalyze the reduction of carbonyl groups to alcohols. A nomenclature system has been proposed for AKRs (227). Like CYPs, AKRs are grouped into families and subfamilies based on amino acid sequence. Families have 40% sequence identity, whereas subfamilies are defined by 60% sequence similarity. The nomenclature consists of the root “AKR” followed by a number for family, a letter for subfamily and finally a number as a unique gene identifier. The nomenclature has also been expanded to accommodate multimers (228). The older (trivial) names of AKRs are still heavily in use, which could lead to confusion. Thus, it is advised to follow the nomenclature system proposed by Jez et al. (227).

There are 15 AKRs in the human genome spanning three families and seven subfamilies. Human AKRs have variable expression and accept a wide range of substrates (**Table 3**). They are involved in the metabolism of sugars, prostaglandins and sterols, as well as the detoxification of carbonyl compounds like lipid peroxidation products. Additionally, members of family 6 (AKR6A3, AKR6A5, and AKR6A9) are constituents of voltage-gated potassium channels (250). All human AKRs have either established or proposed roles in lipid metabolism except for AKR1E2.

**Table 3:** Human AKRs: Genes, expression sites, and example substrates.

Gene	Alternative protein name	Expression sites	Example substrates	Refs.
*AKR1A1*	Aldehyde reductase	Brain, kidney, liver, small intestine	4-HNE, acrolein, succinic semi-aldehyde, D-glucuronic acid, phospholipid aldehydes	(229, 230),
*AKR1B1*	Aldose reductase/Prostaglandin F synthase	Ubiquitous	Glucose, 4-HNE & its glutathione conjugate, acrolein, PGH_2_, phospholipid aldehydes	(229, 230),
*AKR1B10*	Small intestine aldose reductase	Small intestine, colon, liver, cornea	Farnesal, retinoids, acrolein, phospholipid aldehydes	(229, 231–235),
*AKR1B15*	Aldo-keto reductase/3-Keto-acyl CoA reductase	Placenta, testis, adipose tissue	Androgens, estrogens, 3-keto-acyl CoA conjugates	(236)
*AKR1C1*	20α-HSD	Kidney, lung, liver, testis, brain	Progesterone, estrone, 5α-dihydrotestosterone, 4-HNE	(230, 237–239),
*AKR1C2*	Type 3 3α-HSD	Liver, brain, lung, prostate	Progesterone, estrone, 5α-dihydrotestosterone	(237, 238),
*AKR1C3*	Type 5 17β-HSD/Prostaglandin F synthase	Liver, lung, prostate, brain, breast, lymphocytes	Progesterone, estrone, 5α-dihydrotestosterone, PGH_2_, PGD_2_, 4-HNE	(238, 240–242),
*AKR1C4*	Type 1 3α-HSD	Liver	3-Keto-5β-sterols, 5α-dihydrotestosterone	(230, 238),
*AKR1D1*	Steroid 5β-reductase	Liver, placenta, brain	Δ^4^-Ketosteroids, particularly bile acid intermediates	(237, 243–245),
*AKR1E2*	1,5-Anhydro-D-fructose reductase	Liver, testis	1,5-Anhydro-D-fructose	(246, 247)
*KCNAB1*	Potassium voltage-gated channel β-subunit-1 (Kvβ1)/AKR6A3	Brain, heart	Lipid peroxidation-derived aldehydes (presumed)*	(248, 249)
*KCNAB2*	Potassium voltage-gated channel β-subunit-2 (Kvβ2)/AKR6A5	Brain, spinal cord	Methylglyoxal, acrolein, 4-ONE, oxPL, PGJ_2_*	(250–252)
*KCNAB3*	Potassium voltage-gated channel β-subunit-3 (Kvβ3)/AKR6A9	Brain	No data.	(253)
*AKR7A2*	Aflatoxin aldehyde reductase (AFAR1)	Ubiquitous	Aflatoxin B1, succinic semi-aldehyde, 4-HNE	(230, 254)
*AKR7A3*	Aflatoxin aldehyde reductase (AFAR2)	Liver, stomach, pancreas, kidney	Aflatoxin B1	(255),

*Evidence from work on the rat ortholog. 4-HNE, 4-hydroxy-2-nonenal; 4-ONE, 4-oxo-2-nonenal; HSD, hydroxysteroid dehydrogenase; oxPL, oxidized phospholipid; PG, prostaglandin (e.g., PGH_2_, PGD_2_, PGJ_2_).

Human AKRs are generally cytosolic with some exceptions. AKR1B15 is found in mitochondria (236). Members of the AKR1C subfamily in the lung (1C1, 1C2 and 1C3) are also secreted in pulmonary surfactant (256). AKR1B10 is present in lysosomes and is secreted into the intestinal lumen by epithelial cells (257). AKR6 members form a complex with a plasma membrane voltage channel on the cytosolic side. Finally, rat AKR7A2 associates with the Golgi apparatus (258). Human AKR7A2 contains a similar *N*-terminal amphipathic sequence and likely associates with the Golgi apparatus.

#### Mammalian AKRs: Structure and Enzymology

Crystal structures have been solved for many mammalian AKRs. They comprise of a triosephosphate isomerase barrel motif (α/β)_8_ which has alternating α-helices and β-strands repeating eight times. The α-helices surround an internal β-barrel formed by the β-strands. The active site is located at the base of the barrel, in which the substrate and the nicotinamide head group of NADPH (cofactor) are proximally positioned for the reaction, while three flexible loops form the back of the barrel and control substrate specificity (259, 260). The structure also contains two additional helices that are not part of the barrel motif. Family 1 AKRs are monomeric, whereas families 6 and 7 form tetramers and dimers, respectively (258, 261).

AKRs catalyze the stereospecific reduction of carbonyls into alcohols. They can also catalyze the reverse oxidation reaction. However, the reducing direction is generally favored due to the abundance of reduced cofactor in the cellular environment. AKRs can use both NADPH and NADH, but NADPH is the preferred cofactor for most known AKRs as they interact with the phosphoryl group present in NADPH but not NADH (262, 263). The stereospecific nature of the reaction is due to the orientation of the NADPH and the substrate in the active site. The NADPH is bound in the anti-conformation (with respect to the ribose ring) which promotes the transfer of the 4-*pro*-R hydride (264), and the substrate is positioned perpendicular to the cofactor.

**Figure 4:**
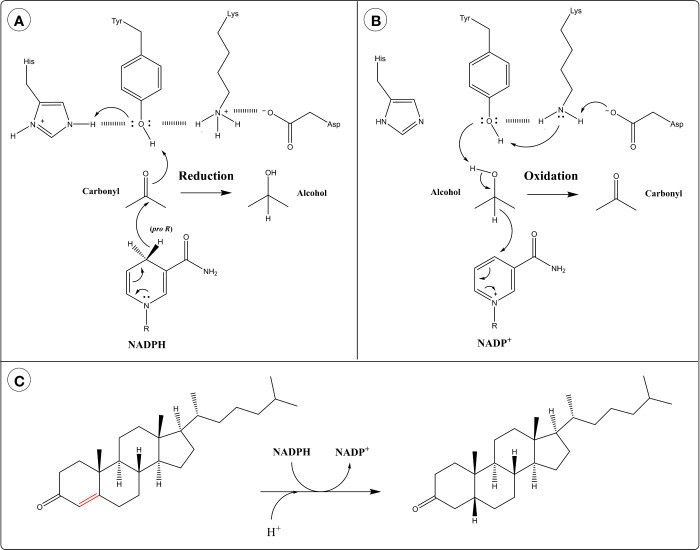
Catalytic mechanism of aldo-keto reductases (AKRs) in the: **(A)** Reduction direction and **(B)** Oxidation direction. **(C)** 5β-Reduction of steroid double bond by AKR1D1.

The reaction of AKRs follows an ordered bi-bi mechanism in which the NADPH cofactor binds first and leaves last. The active site of AKRs contains a highly conserved catalytic tetrad of Tyr, Lys, Asp and His. The hydride transfer is facilitated by acid-base catalysis involving the protonation state of Tyr and the other catalytic residues. In the reduction direction, the protonated form of Tyr acts as an acid by participating in proton relay with His (259) (**Figure 4A**). This polarizes the carbonyl group of the substrate, allowing it to accept a hydride ion from the cofactor. In the oxidation direction, the phenolate form of Tyr (generated through proton relay with Lys and Asp) acts as a base and abstracts a proton from the alcohol group of the substrate, facilitating hydride transfer to the cofactor (259) (**Figure 4B**).

AKR1D subfamily members catalyze the irreversible 5β-reduction of sterol double bonds into single bonds, instead of the reversible reduction of carbonyls typical of AKRs (**Figure 4C**). They possess an altered catalytic tetrad with Glu replacing His (265). This substitution allows the substrate to penetrate deeper into the active site pocket, positioning C5 close to the 4-*pro*-H of NADPH (259). The catalytic Glu residue is thought to act as a superacid, facilitating the enolization of the steroid double bond and allowing hydride transfer to C5 (259, 265).

Human AKR1B1 catalyzes the isomerization of PGH_2_ to PGD_2_ in the absence of cofactor, as well as the reduction of PGH_2_ to PGF_2α_ in the presence of cofactor (266). Curiously, both of these reactions are reported to be facilitated by a catalytic triad of Lys, His and Asp, without the involvement of Tyr in catalysis, although Tyr is still required for the *p*‐nitrobenzaldehyde reductase activity of the enzyme (266). Thus, both Tyr and His can participate in acid-base catalysis, at least in AKR1B1.

#### Involvement of Human AKRs in Lipid Metabolism

Human AKRs play roles in the detoxification of reactive carbonyls. Several AKRs metabolize lipid peroxidation-derived aldehydes like acrolein and 4-HNE (**Table 3**). Additionally, some AKRs (1A1, 1B1, 1B10, 6A5) can reduce oxidized phospholipids, particularly phospholipid aldehydes (229, 251). These activities mitigate the cytotoxicity of reactive carbonyls (240, 267–269), and in the case of family 6 AKRs are also thought to function in redox sensing (251).

Both AKR1B1 and AKR1C3 exhibit prostaglandin F synthase activity (270, 271). AKR1B1 catalyzes the reduction of PGH_2_ to PGF_2α_. AKR1C3 catalyzes the same reaction as well as the reversible reduction of PGD_2_ to 9α,11β-PGF_2_ (241). Furthermore, AKR1B1 catalyzes the isomerization of PGH_2_ to PGD_2_ exclusively in the absence of NADPH as noted previously. However, the biological relevance of this activity is not clear as this isomerization reaction is also catalyzed by two prostaglandin D synthases (272), and the cellular redox status favors the reduction reaction. Both PGF_2α_ and its isomer 9α,11β-PGF_2_ promote uterine contractions during labor (237).

Six of the 15 human AKRs are involved in sterol/steroid metabolism: AKR1B15, the four members of the AKR1C subfamily (also grouped as hydroxysteroid dehydrogenases; HSDs), and AKR1D1. AKR1B15 catalyzes the 17β-reduction of androgens and estrogens (236). AKR1C1 (20α-HSD) catalyzes the 20α-reduction of progesterone inactivating it (273), whereas AKR1C2 (Type 3 3α-HSD) possesses a 3α-dehydrogenase activity and deactivates 5α-dihydrotesterone (259). AKR1C3 (Type 5 17β-HSD) exhibits 17-ketoreductase activity and produces testosterone and estradiol from their respective precursors.

AKR1D1 and AKR1C4 (Type 1 3α-HSD) catalyze key steps in bile acid biosynthesis. AKR1D1 catalyzes irreversible 5β-reduction of a double bond in 3-ketosterols. This modification changes the geometry of the steroid nucleus from flat to twisted, with a bend in the A/B ring junction. Next, AKR1C4 catalyzes the 3α-reduction of the ketosteroid, resulting in a 3α,5β-configuration, a characteristic feature of bile acids.

#### Other HSDs Belong to the Short-Chain Dehydrogenase/Reductase (SDR) Family

Some HSDs belong to a different family of enzymes: the short-chain dehydrogenases/reductases (SDR). SDRs exhibit key differences from AKRs in structure, kinetics, catalytic mechanism, and reaction stereochemistry (reviewed in (274)). Of note, HSDs of the SDR family work as either ketosteroid reductases or hydroxysteroid oxidases, depending on their preference for the corresponding forms of NADP(H) or NAD(H). This contrasts with HSDs of the AKR1C family which operate in the reduction direction using (primarily) NADPH (274). Like CYPs and AKRs, a systematic nomenclature has been proposed for SDRs (275). However, the old names (especially for HSDs) remain heavily in use. HSDs of the SDR family have important functions in sterol/steroid metabolism (**Table 4**).

**Table 4:** Human SDR-HSDs and their substrates/reactions.

Gene	Alternative protein names	Substrates	Reaction	Refs.
*HSD3B1*	Type 1 3β-HSD/Δ^5-4^ isomerase/SDR11E1	3β-Hydroxy-Δ^5^-sterols	Oxidation & isomerization	(276, 277),
*HSD3B2*	Type 2 3β-HSD/Δ^5-4^ isomerase/SDR11E2	3β-Hydroxy-Δ^5^-sterols	Oxidation & isomerization	(277, 278),
*HSD3B7*	Type 7 3β-HSD/SDR11E3	3β-Hydroxy-Δ^5^-sterols	Oxidation & isomerization	(279)
*HSD11B1*	Type 1 11β-HSD/Corticosteroid 11β-dehydrogenase isozyme 1/SDR26C1	11β-Hydroxysterols (e.g. cortisol),	Oxidation	(280)
*HSD11B2*	Type 2 11β-HSD/Corticosteroid 11β-dehydrogenase isozyme 2/SDR9C3	11β-Hydroxysterols (e.g. cortisol),	Oxidation	(280)
*HSD17B1*	Type 1 17β-HSD/Estradiol 17β-dehydrogenase 1/SDR28C1	Estrogens and androgens	Reduction	(281, 282)
*HSD17B4*	Type 4 17β-HSD/Peroxisomal multifunctional enzyme type 2/SDR8C1	(24R,25R)-3α,7α,12α,24-Tetrahydroxy-5β-cholestan-26-oyl-CoA	Reduction	(283)
*HSD17B7*	Type 7 17β-HSD/3-Ketosteroid reductase/SDR37C1	3β-Hydroxysterols, 17β-estradiol	Reduction	(284, 285)

## Biosynthetic Pathways of Oxidized Lipids

### Oxygenated PUFA and Oxidized Phospholipids

#### Classification of Oxygenated PUFA as Eicosanoids and Docosanoids

The term “oxylipin” refers to oxygenated PUFA derivatives. It encompasses a wide range of oxidized lipids like hydroxy-, epoxy-, oxo-FAs, and endoperoxides. Oxylipins can be classified according to their precursor into eicosanoids (C20) or docosanoids (C22), etc., and this system will be used from here on. Following the classification system of the LIPID MAPS consortium, eicosanoids include prostaglandins, leukotrienes, thromboxanes, lipoxins, hepoxilins, E-resolvins, as well as hydroxy-, hydroperoxy-, epoxy-, and oxo-eicosanoids (286). Likewise, docosanoids include D-resolvins, protectins, maresins, hydroxy-, hydroperoxy-, epoxy-, and oxo-docosanoids. Structures of these lipids are available on the LIPID MAPS database (287).

Here, we focus on the biosynthesis of eicosanoids derived from AA and EPA, and docosanoids derived from DHA, as they are the most studied. Due to the broad substrate specificity of enzymes involved in lipid oxidation, PUFAs with shorter chains or different number of double bonds also undergo similar reactions. Similarly, oxygenation of endocannabinoids occurs on the PUFA moiety and follows the enzymatic mechanisms described here. Further aspects of oxygenated endocannabinoids are discussed in other reviews (216, 288).

#### Release of PUFA from Membranes for Oxylipin Biosynthesis

Intracellular concentration of free PUFA is tightly regulated through conjugation with coenzyme A (CoA) and subsequent esterification into lysoPL forming PL or shuttling into other pathways (like β-oxidation). PUFAs are abundant in membrane PLs, typically esterified at the *sn-*2 position. The classical pathway of oxylipin biosynthesis involves the release of PUFA from membrane PL *via* the action of lipases like phospholipase A_2_ (PLA_2_), phospholipase C and diacylglycerol lipase (289, 290). PLA_2_ enzymes are classified into six types: secreted (sPLA_2_), cytosolic (cPLA_2_), calcium-independent (iPLA_2_), platelet-activating factor acetylhydrolases (PAH-AH), lysosomal (LPLA_2_), and adipose (AdPLA), and these are further divided into groups and subgroups (291, 292). cPLA_2_α is localized in the cytosol but translocates to intracellular membranes upon calcium activation, and exhibits selectivity for phospholipids containing AA at the *sn*-2 position (293, 294). On the other hand, the release of DHA from the membrane can be facilitated by iPLA_2_β (295, 296). Last, sPLA_2_ enzymes are involved in the release of AA, EPA and DHA (297, 298). PLA_2_ enzymes are extensively reviewed by Murakami (299).

#### Biosynthesis of Eicosanoids

**Figure 5:**
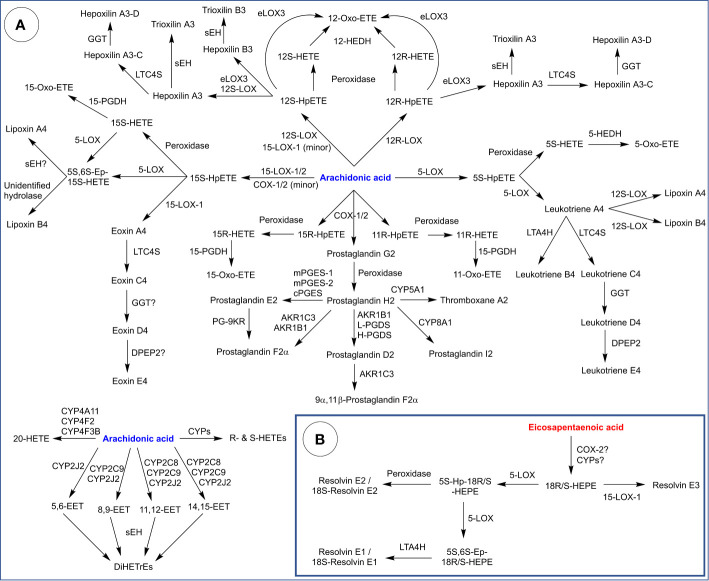
Biosynthesis of eicosanoids derived from: **(A)** Arachidonic acid and **(B)** Eicosapentaenoic acid. HETE, hydroxy-eicosatetraenoic acid; Hp, hydroperoxy; Ep, epoxy; LTA4H, leukotriene A_4_ hydrolase; LTC4S, leukotriene C_4_ synthase; GGT, γ-glutamyl transpeptidase; DPEP2, dipeptidase 2; sEH, soluble epoxide hydrolase; 12-HEDH, 12-hydroxyeicosanoid dehydrogenase; PGES, prostaglandin E synthase (m: microsomal, c = cytosolic); PGDS (prostaglandin D synthase (L: lipocalin type, H: hematopoietic); 15-PGDH, 15-hydroxyprostaglandin dehydrogenase; PG-9KR, prostaglandin 9-ketoreductase; EET, epoxyeicosatrienoic acid; DiHETrE, dihydroxy-eicosatrienoic acid; HEPE, hydroxy-eicosapenataenoic acid.

Arachidonic acid is a substrate of eicosanoids produced through the actions of COXs, LOXs, CYPs, and other downstream enzymes (**Figure 5A**). The most established of these are mono-oxygenation products of AA, DHA, and EPA and classic prostaglandins, leukotrienes, and thromboxane from COXs, LOXs, and CYPs. Most of these were described from the 1950s–90’s in seminal studies, with many conducted at the Karolinska Institute in Stockholm by Sune Bergstrom, Bengt Samuelsson and colleagues. Indeed, the Nobel Prize for Physiology and Medicine was awarded for discovery of prostaglandins and related biologically active substances in 1985 to Bergstrom, Samuelsson and John Vane. More recently, research has focused on characterization of multiply oxygenated PUFAs from AA, EPA, and DHA including several which are proposed to form from transcellular sequential oxygenation by various enzymes. However, for many of these, their specific biosynthetic pathways, enantiomeric composition and biological actions in tissues are not well understood and the levels formed are extremely low in comparison to classic PGs and monohydroxy-oxylipins. Furthermore, their bioactions often require amounts of lipids that are considerably higher than levels detected in cell and tissue samples, and thus could be considered pharmacological.

COXs convert AA into PGG_2_ which is reduced into PGH_2_ (**Figure 5A**). From there, PGH_2_ functions as substrate for classic PGs and thromboxane, generated by tissue-specific enzymes. CYP5A1 (thromboxane synthase) is expressed in several cell types and tissues (platelets, macrophages, lung, kidney, liver) and catalyzes the isomerization of PGH_2_ into thromboxane A_2_, a potent vasoconstrictor and activator of platelet aggregation (300, 301). CYP5A1 also catalyzes the cleavage PGH_2_ into malondialdehyde and 12-hydroxyheptadecatrienoic acid. CYP8A1 (prostacyclin synthase) is widely expressed (abundant in ovary, heart, lung, skeletal muscle, and prostate) and catalyzes the isomerization of PGH_2_ into prostacyclin (PGI_2_), a vasodilator and inhibitor of platelet aggregation (302).

PGH_2_ can also be converted into PGD_2_ through the action of AKR1B1 or PGD synthases, of which there are two isoforms: lipocalin-type PGD synthase (in the central nervous system, male genitalia, heart, cerebrospinal fluid and plasma) and hematopoietic PGD synthase (in antigen-presenting cells, mast cells and megakaryocytes) (266, 303). PGD_2_ plays roles in the regulation of body temperature, sleep cycle, pain perception and the immune response (304). In the uterus, AKR1C3 catalyzes the reduction of PGD_2_ into 9α,11β-PGF_2_, which promotes uterine contractions (237). Similarly, AKR1C3 and AKR1B1 catalyze the reduction of PGH_2_ into PGF_2_α, which also promotes uterine contractions (305). Finally, PGH_2_ can be converted into PGE_2_ *via* the action of PGE synthases, of which there are three isoforms: an inducible microsomal isoform (mPGES-1), a constitutive microsomal isoform (mPGES-2) and a constitutive cytosolic isoform (cPGES) (306). PGE_2_ is abundant in the body and plays a complex role in immunity and inflammation (307). Additionally, PGE_2_ is reduced into PGF2α by prostaglandin 9-ketoreductase (PG-9KR) (308).

COXs also produce 11*R*-, 15*R*-, and 15*S*-HpETEs as side products, all three of which can be reduced into their corresponding alcohols by peroxidase activity, which are in turn reduced into oxo-ETEs by 15-hydroxyprostaglandin dehydrogenase (15-PGDH). Bioactivities of HETEs and oxo-ETEs are extensively reviewed by Powell and Rokach (309).

5-LOX converts AA into 5*S*-HpETE (**Figure 5A**), which is reduced into 5*S*-HETE through the action of a peroxidase (e.g., GPX) or converted into leukotriene A_4_ (LTA_4_) through the leukotriene synthase activity of 5-LOX. Various immune cells (neutrophils, monocytes, platelets) express 5-hydroxyeicosanoid dehydrogenase (5-HEDH) which converts 5*S*-HETE into 5-oxo-HETE, a potent chemoattractant for eosinophils (310). On the other hand, LTA_4_ serves as a precursor for LT peptide conjugates (LTC_4_, D_4_ and E_4_) and lipoxins (A and B). LTC_4_, D_4_, and E_4_ are synthesized from LTA_4_ through LTC_4_ synthase (a gluthathione-*S*-transferase), γ-glutamyl transpeptidase (GGT) and dipeptidase 2 (DPEP2). Leukotrienes exhibit pro-inflammatory properties and are implicated in asthma and allergic reactions (311). On the other hand, lipoxins are thought to be synthesized by 12S-LOX through a transcellular pathway which involves interactions between two cell types (312). Lipoxins exhibit anti-inflammatory properties and are proposed to play a role in wound healing and tissue homeostasis (313). Alternatively, LTA_4_ can be hydrolyzed by leukotriene A_4_ hydrolase (LTA4H) into LTB_4_, which attracts neutrophils (314).

15-LOXs converts AA into 15*S*-HpETE (**Figure 5A**), which can be reduced into 15S-HETE then oxidized into 15-oxo-ETE. 15*S*-HpETE and 15*S*-HETE are also substrates for 5-LOX, which produces a 5*S*,6*S* epoxy intermediate that can be hydrolyzed into lipoxins by unidentified hydrolases. Additionally, 15*S*-HpETE is proposed to be a precursor for eoxin A_4_ (a 14,15 leukotriene), which is converted into peptide conjugates (eoxin C_4_, D_4_, and E_4_) similar to leukotrienes (315). Conversion of eoxin A_4_ into C_4_ is thought to involve LTC4S. Enzymes that catalyze the following two steps leading to eoxin D_4_ and E_4_ are unidentified, presumably a GGT isoform and DPEP2. Eoxins are produced in eosinophils, mast cells and airway epithelial cells, and have been shown to possess pro-inflammatory properties (316).

12*S*-HpETE and 12*R*-HpETE are produced by 12S-LOX and 12*R*-LOX, respectively (**Figure 5A**). These hydroperoxides can be reduced to their corresponding alcohols *via* a peroxidase, and both alcohols further oxidized into 12-oxo-ETE by 12-hydroxyeicosanoid dehydrogenase (12-HEDH) (309). Both hydroperoxides are also precursors for hepoxilins, which are epoxy-alcohols. eLOX3 in skin converts 12*R*-HpETE into either hepoxilin A_3_ or 12-oxo-ETE. On the other hand, 12*S*-HpETE can be converted into hepoxilin A_3_ or B_3_ by either 12S-LOX or eLOX3, but eLOX3 also generates 12-oxo-ETE (70, 317). Hepoxilins A_3_ and B_3_ are hydrolyzed by sEH into trioxilins A_3_ and B_3_, respectively (318). Hepoxilin A_3_ can also be conjugated with glutathione *via* LTC4S producing hepoxilin A_3_-C, which can be converted into a hepoxilin A_3_-D by GGT (319). The occurrence of hepoxilin A_3_-E (cysteinyl conjugate) has also been proposed but not confirmed. Hepoxilin A_3_ is proposed to regulate mucosal inflammation by recruiting neutrophils across the epithelial junction into the gut lumen (320).

CYPs generate various midchain *R*- and *S*-HETEs as well as 20-HETE through hydroxylation of AA (**Figure 5A**). Alternatively, epoxyeicosatrienoic acids (EET) are produced from AA through epoxidation of its double bonds. Major mammalian CYP epoxygenases include CYP2C8, CYP2C9, and CYP2J2 (15). EETs are further metabolized by sEH (and potentially other epoxide hydrolases) into dihydroxyeicosatrienoic acids (DiHETrE). Note that EPA and other PUFA are also targets of CYP-mediated reactions, which generate the corresponding hydroxy-, epoxy-, and dihydroxy- derivatives.

“E-resolvins”, another class of eicosanoids, are proposed to be generated from EPA (**Figure 5B**). COX-2 and CYPs have both been proposed to act on EPA to produce 18*R*-hydroxyeicosapentaenoic acid (18*R*-HEPE), which is in turn a proposed precursor for lipids termed resolvins E1, E2 and E3 (E-resolvins). It is known that the acetylation of COX-2 shifts its activity into favoring the lipoxygenase-type reaction over the cyclooxygenase activity, and that acetylated COX-2 generates 18*R*-HEPE from EPA (321). However, acetylated COX-2 is not a physiologically relevant form, and 18*R*-HEPE could be potentially generated through other means. COX-2 is theoretically capable of generating 18*R*-HEPE from EPA in a lipoxygenase-type reaction followed by peroxidase activity. However, whether the generation of 18*R*-HEPE from EPA occurs *in vivo* and in sufficient amounts without COX-2 acetylation is unknown. Microbial CYPs have also be proposed as sources of 18*R*-HEPE during infection. E-resolvins are proposed to be produced through a transcellular mechanism involving microbial and mammalian cells (322). Alternatively, mammalian CYPs are also potential sources of 18*R*-HEPE, although the exact isoforms involved are unknown.

18*R*-HEPE can be metabolized by 5-LOX into a 5S-hydroperoxy intermediate and further into a 5*S*,6*S*-epoxy intermediate (323). The former is proposed to be converted into resolvin E2 by a peroxidase, whereas the latter is proposed to be hydrolyzed into resolvin E1 by LTA4H (324). 18*S*-analogues of resolvin E1 and E2 have also been described and are proposed to be generated from 18*S*-HEPE through a similar pathway (**Figure 5B**), although the enzymatic sources of 18*S*-HEPE (aside from acetylated COX-2) are also unknown. 18*R*-HEPE is also proposed to be a precursor to two stereoisomers collectively called resolvin E3 generated by 15-LOX-1 (325). E-resolvins are proposed to exhibit anti-inflammatory effects and promote the resolution of inflammation (325, 326).

Both lipoxins and E-resolvins have been classified as specialized pro-resolving mediators (SPMs), a term which currently describes families of oxygenated PUFA metabolites with proposed roles in the resolution of inflammation and tissue regeneration generated at extremely low levels in biological samples.

#### Biosynthesis of Docosanoids

**Figure 6:**
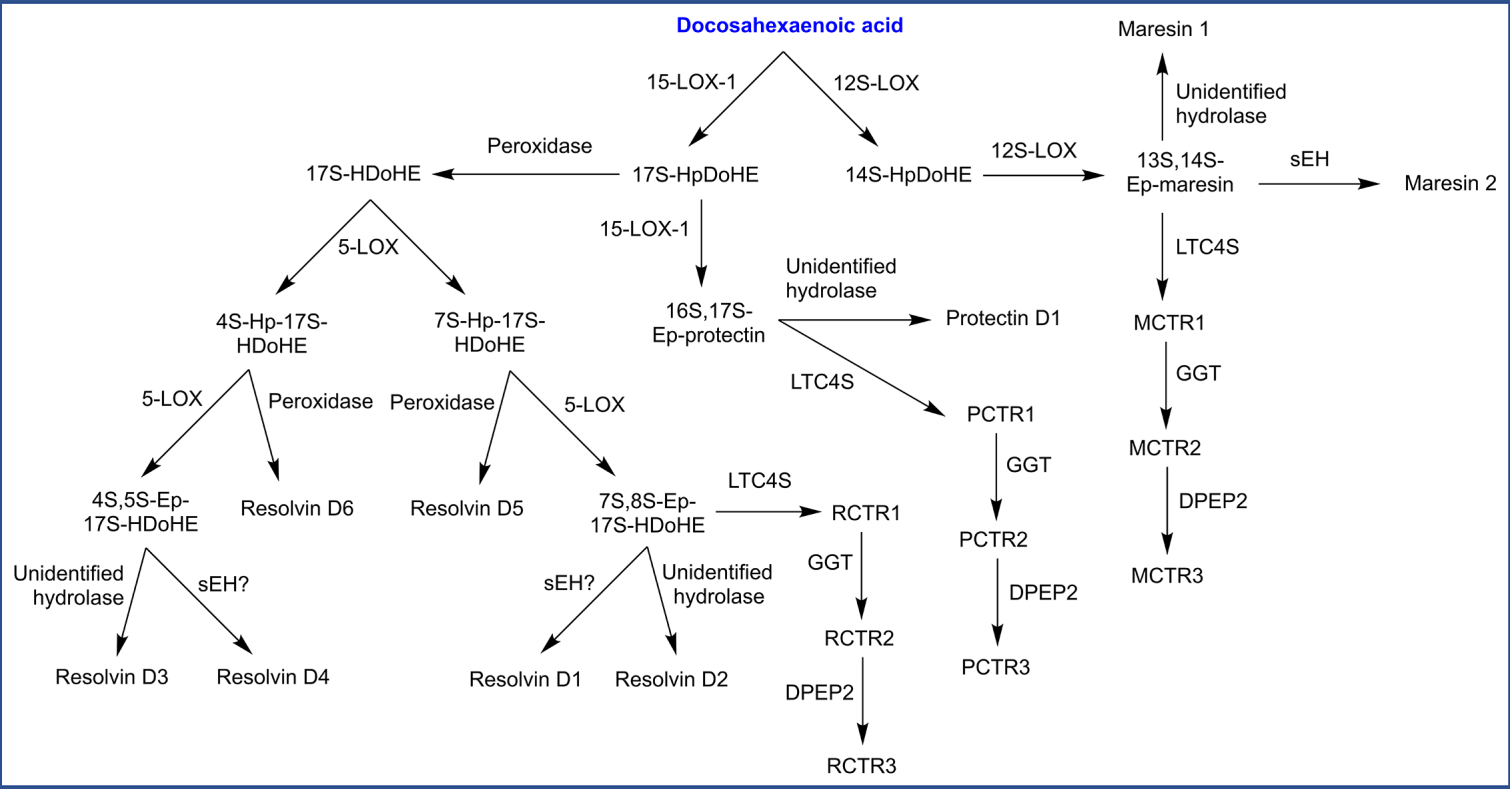
Biosynthesis of specialized pro-resolving mediators (SPMs) derived from docosahexaenoic acid. HDoHE, hydroxy-docosahexaenoic acid; Hp, hydroperoxy; Ep, epoxy; CTR, conjugate in tissue regeneration (M: maresin, P: protectin, R: resolvin); sEH, soluble epoxide hydrolase; LTC4S, leukotriene C_4_ synthase; GGT, γ-glutamyl transpeptidase; DPEP2, dipeptidase 2.

Oxygenation of DHA by LOXs and CYPs forms oxygenated docosanoids (hydroxy-, epoxy-, and dihydroxy derivatives). DHA is also the precursor to several classes of multiply oxygenated docosanoids, including additional SPMs which have been named maresins, D-resolvins, and protectins (**Figure 6**). Maresin biosynthesis has been proposed to start with action of 12S-LOX, generating 14*S*-hydroperoxy-DHA (14*S*-HpDoHE) then a 13*S*,14*S*-epoxy intermediate. Hydrolysis of this epoxy intermediate by sEH or an unidentified hydrolase is proposed to generate maresin 2 and maresin 1, respectively (93, 327). The biosynthesis of protectin and D-resolvins both begin with 15-LOX-1 which generates a 17*S*-hydroperoxide (17*S*-HpDoHE). Further conversion of this hydroperoxide into a 16*S*,17*S*-epoxy intermediate by 15-LOX-1 followed by hydrolysis (unidentified hydrolase) is proposed to generate protectin D1 (328). Alternatively, 17*S*-HpDoHE could be reduced into an alcohol by a peroxidase, which then serves as a precursor for D-resolvins. In this route, 5-LOX is proposed to generate two distinct products hydroperoxides (4*S* or 7*S*), which undergo further transformations leading to several D-resolvins (**Figure 6**). Additionally, epoxide intermediates in the previously described pathways are proposed to undergo glutathione conjugation by LTC4S, generating peptide conjugates of maresin, protectin and D-resolvin named MCTR1, PCTR1, and RCTR1, respectively (CTR = conjugate in tissue regeneration). These conjugates are proposed to be metabolized by GGT and DPEP2, similar to leukotrienes (329).

#### Oxygenation of Esterified PUFA and Biosynthesis of Oxidized Phospholipids

The classical pathway for the biosynthesis of oxidized phospholipids starts with the release of PUFA from membranes through the action of PLA_2_. PUFA are then oxygenated by the enzymes discussed earlier. This is followed by acylation of oxygenated PUFA with CoA and esterification into a lysoPL *via* the action of an *sn*-2 acyltransferase.

The generation of PL-esterified eicosanoids is well-documented in mammalian cells such as epithelial, endothelial and immune cells (330). HETE-PLs are acutely generated by activated neutrophils, platelets and monocytes (331–333). Similarly, PGD_2_ and PGE_2_ generated from COX-1-derived PGH_2_ in activated platelets are rapidly incorporated into PE-lysoPLs (334). Also, EET-PLs have been detected in rat liver (335). For most of these, the mechanism of formation requires endogenous generation of an oxylipin, which is then rapidly esterified into membrane PL pools (e.g., on a timescale of minutes) *via* Lands cycle enzymes (336).

15-LOXs also contribute to the formation of oxPL by two other pathways. The first involves direct oxygenation of membrane PLs (331, 337). In the second pathway, 15-LOXs oxygenate the PUFA moiety of CEs, then hydrolysis of oxygenated PUFA from CE liberates oxygenated PUFA which can be esterified to lysoPL (87).

**Figure 7:**
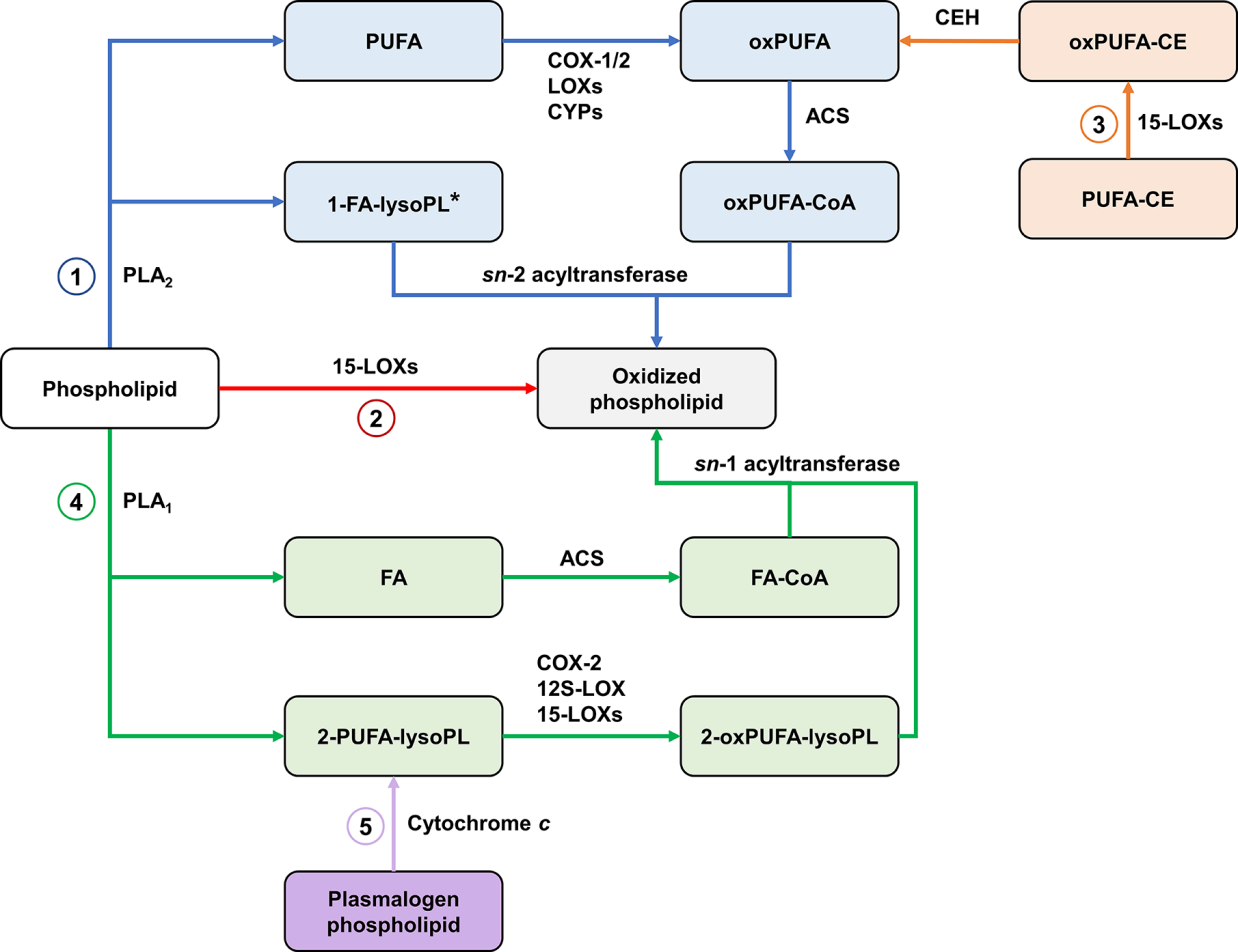
Biosynthetic pathways of oxidized phospholipids. **(1)** The classical pathway involves the action of PLA_2_ on membrane phospholipids, releasing *sn*-2 PUFA which are oxygenated by cyclooxygenases (COXs), lipoxygenases (LOXs), and cytochrome P450s (CYPs) then re-esterified. *Oxygenated PUFA can be also be esterified with plasmalogen lysophospholipids **(2)** Direct oxygenation of membrane phospholipids by 15-LOXs. **(3)** 15-LOX-mediated oxygenation of PUFA in cholesteryl esters followed by hydrolysis of oxygenated PUFA provides substrates for the classical pathway. **(4)** An alternative pathway involves the action of PLA_1_, forming 2-PUFA-lysophospholipids which are oxygenated by COX-2, 12S-LOX, and 15-LOXs then re-esterified with FA. **(5)** Cytochrome *c* releases 2-PUFA-lysophospholipids by cleaving the vinyl ether bond in plasmalogen phospholipids, providing substrates for the alternative pathway. PLA, Phospholipase A; oxPUFA, oxygenated PUFA; ACS, acyl-CoA synthase; CE, cholesteryl ester; CEH, neutral cholesterol ester hydrolase; FA, fatty acid/acyl; lysoPL, lysophospholipid.

Recent studies found that COX-2, 15-LOX-2, and platelet 12S-LOX can catalyze the oxygenation of 2-AA-lysoPL released by iPLA_2_γ in response to calcium ionophore stimulation (88, 94). These reactions generate 2-eicosanoid-lysoPLs which are proposed to be a source of free eicosanoids as well as acting as signaling mediators themselves. 2-eicosanoid-lysoPLs can also be converted into oxPLs through the action of *sn*-1 acyltransferase (338). Cytochrome *c* has been recently been identified as a plasmalogenase, and is proposed to be a source of 2-eicosanoid-lysoPLs under conditions of oxidative stress (339). These findings describe novel pathways for the biosynthesis of oxPLs (**Figure 7**). Further research is required to assess the contribution of these pathways to the formation of oxPLs and other mediators *in vivo*.

### Biosynthesis of Oxysterols, Bile Acids, and Steroid Hormones

#### Oxysterols

**Figure 8:**
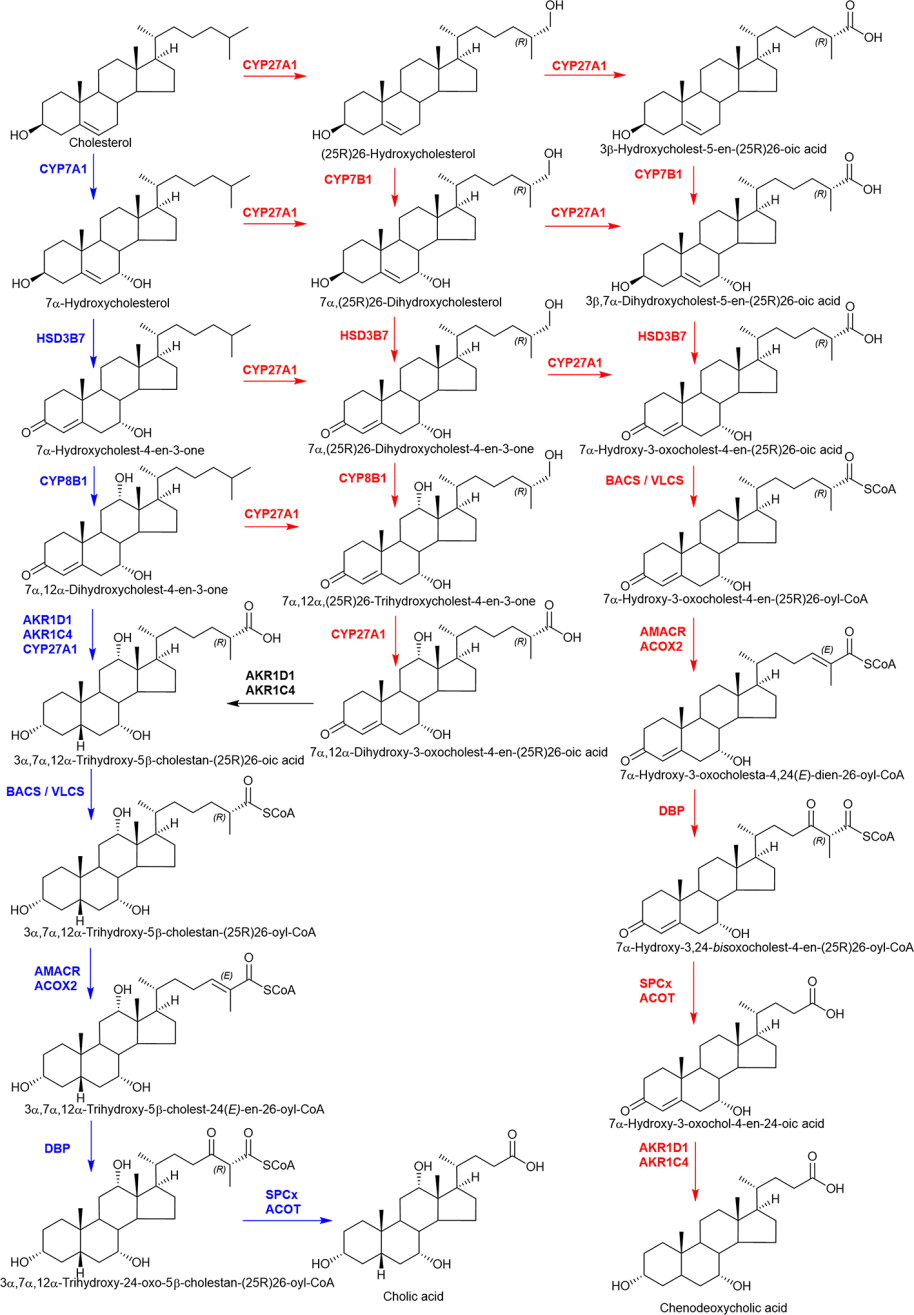
A simplified view of the major bile acid biosynthesis pathways. The “neutral” pathway (highlighted in blue) starts with 7α-hydroxylation of cholesterol by CYP7A1, the “acidic” pathway with (25R)26-hydroxylation then (25R)26-carboxylation of cholesterol by CYP27A1. In the “acidic” pathway (highlighted in red) CYP7B1 is the 7α-hydroxylase.

**Figure 9:**
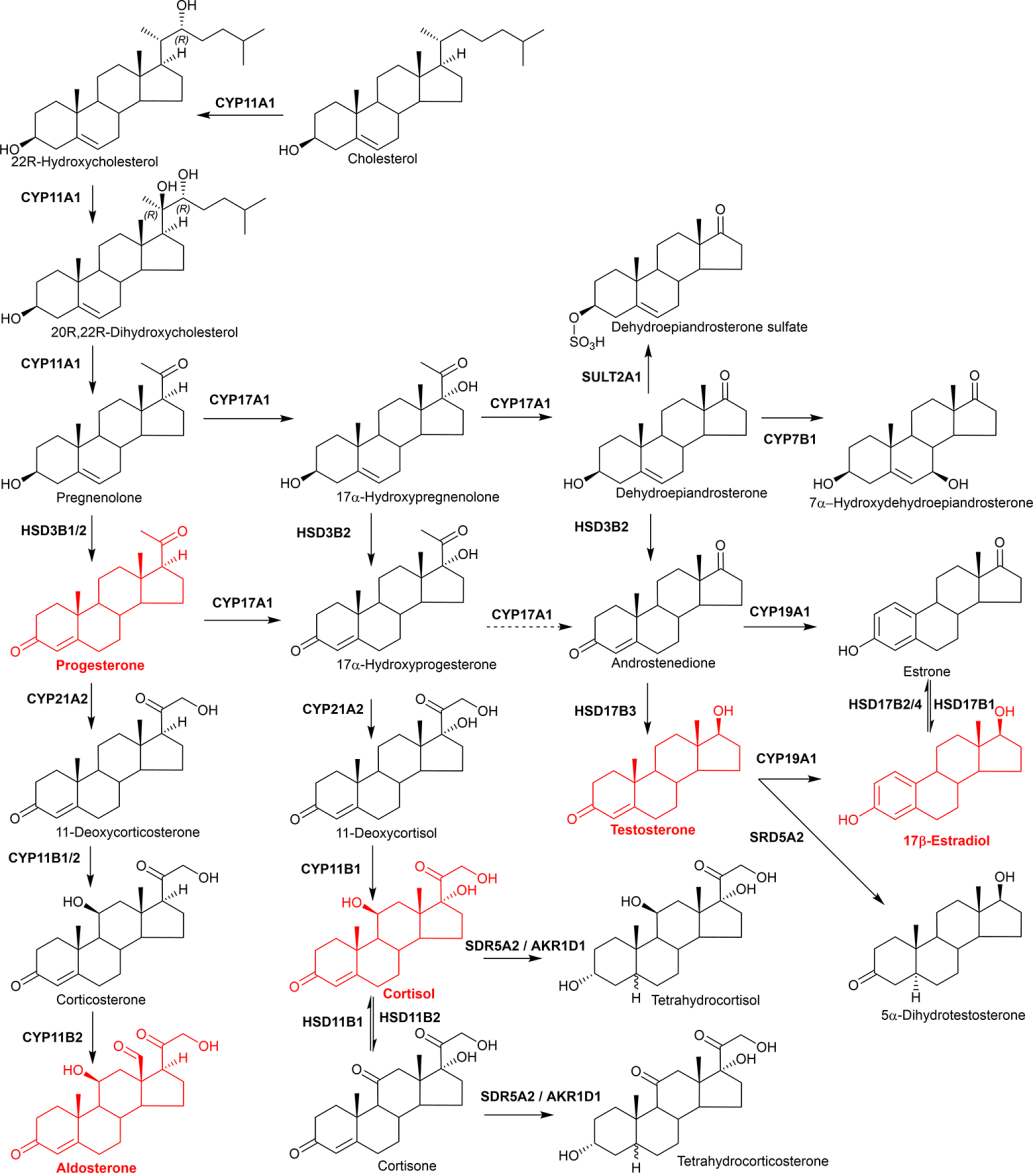
Simplified view of steroid hormone biosynthesis. Highlighted in red are the classical steroid hormones, progesterone, aldosterone, cortisol, testosterone, and 17β-estradiol.

Oxysterols are formed in the first steps of cholesterol metabolism: they are oxidized forms of cholesterol and also of its precursors (340). 7α-Hydroxycholesterol (7α-HC) is formed from cholesterol by CYP7A1 and represents the first metabolite in the neutral pathway of bile acid biosynthesis (**Figure 8**) (219, 220). (25R)26-Hydroxycholesterol (26-HC), more commonly called 27-hydroxycholesterol, and 3β-hydroxycholest-5-en-(25R)26-oic acid (3β-HCA) are both formed from cholesterol by CYP27A1 and are the first members of the acidic or alternative pathway of bile acid biosynthesis (219–221). While CYP7A1 is an endoplasmic reticulum and liver specific protein, CYP27A1 is mitochondrial and expressed in many tissues. CYP46A1 is almost exclusively expressed in neurons, its function is to maintain cholesterol balance in the brain, converting cholesterol from a molecule unable to pass the blood brain barrier to 24S-hydroxycholesterol (24S-HC), a more polar molecule which can cross the barrier (222, 223, 341). CYP11A1, like CYP27A1, is a mitochondrial inner membrane protein. It is highly expressed in steroidogenic tissue and will oxidize cholesterol to pregnenolone in a three step process involving 22R-hydroxycholesterol (22R-HC) and 20R,22R-dihydroxycholesterol as intermediates (20R,22R-diHC) (342, 343) (**Figure 9**). The pathways of bile acid biosynthesis are discussed in more detail below.

While most “primary” oxysterols are formed from cholesterol in CYP catalyzed reactions, cholesterol 25-hydroxylase (CH25H), the dominating enzyme that generates 25-hydroxycholesterol (25-HC) is an exception, in that is not a CYP, but a member of a family of enzymes that utilize di-iron cofactors to catalyze the hydroxylation (344). CH25H is expressed in activated immune cells and 25-HC has both anti-bacterial and anti-viral activities (345–348). 25-HC is metabolized by CYP7B1 to 7α,25-diHC, a ligand to the GPCR Epstein Barr virus induced gene 2 (EBI2 or GPR183). 7α,25-diHC acts as a chemoattractant to GPR183 expressing immune cells (349, 350). An alternative route to 7α,25-diHC production is through CYP3A4 oxidation of 7α-HC (190), while the same enzyme has also been reported to act as a second cholesterol 25-hydroxylase and also a 4β-hydroxylase of cholesterol (189, 351).

Oxysterols can also be formed from cholesterol precursors in reactions catalyzed by CYP enzymes (202, 352). These reactions may be important in patients suffering from inborn errors of cholesterol biosynthesis such as Smith-Lemli-Opitz syndrome (SLOS, 7-dehydrocholesterol reductase deficiency) and desmosterolosis (3β-hydroxysterol-Δ^24^-reductase deficiency) or where there is very high expression of sterol hydroxylases, e.g., CYP7A1 in cerebrotendinous xanthomatosis, where CYP27A1 is deficient.

#### Bile Acid Biosynthesis

There are two quantitatively major and at least five minor pathways of bile acid biosynthesis and all pathways involve multiple oxidation reactions (219, 220, 353–358). Besides CYP enzymes, key oxidation reactions are carried out by hydroxysteroid dehydrogenase (HSD) members of the short chain dehydrogenase/reductase (SDR) family and reductions by aldo-keto reductases (AKR) enzymes. One of the minor pathways that results in the formation of 3β,5α,6β-trihydroxycholan-24-oic acid involves of cholestane-3β,5α,6β-triol which is likely formed *via* the cholesterol peroxidation product 5,6-epoxycholesterol (219, 354).

While the acidic pathway may be most important in infants (359), at later stages of life the neutral pathway is dominant. This is initiated by CYP7A1 oxidation of cholesterol to generate 7α-HC in the liver. Further oxidation may occur at C-12 by CYP8B1 to generate 7α,12α-dihydroxycholesterol (7α,12α-diHC), which may be preceded or succeeded by oxidation and isomerization of the 3β-hydroxy-5-ene structure to a 3-oxo-4-ene by HSD3B7, giving 7α-hydroxycholest-4-en-3-one (7α-HCO) and 7α,12α-dihydroxycholest-4-en-3-one (7α,12α-diHCO), respectively (**Figure 8**). A general feature of bile acid biosynthesis is that many of the enzymes involved in the pathways accept multiple substrates resulting in variations in the order of reactions depending on the tissue in which they proceed (360). The next steps involve A-ring reductions which may be succeeded or preceded by (25R)26-hydroxylation and (25R)26-carboxylation to ultimately give 3α,7α,12α-trihydroxy-5β-cholanestan-(25R)26-oic acid. The A-ring reductions are carried out by AKR1D1 and AKR1C4, while CYP27A1 carries out the (25R)26-oxidations. Side-chain shortening of the cholestanoic acid proceeds in the peroxisome through the CoA-thioester formed by bile acid Co-A synthetase (BACS, *SLC27A5*) or very long chain acyl-CoA synthetase (VLCS, *SLC27A2*). Following C-25 racemization by α-methylacyl-CoA racemase (AMACR) the next oxidation involves the introduction of Δ^24^ double bond by the enzyme acyl-CoA oxidase 2 (ACOX2). The Δ^24^ double bond is then hydrated by D-bifunctional protein (DBP), which then oxidizes the C-24 hydroxy to a C-24 ketone *via* HSD17B4 activity. The resulting product 3α,7α,12α-trihydroxy-24-oxo-5β-cholestan-(25R)26-oyl-CoA is then oxidized by the enzyme peroxisomal thiolase 2 (SPCx) to the thioester of cholic acid ready for conjugation with glycine, taurine, or hydrolysis to the free acid by peroxisomal acyl-CoA thioesterase (ACOT) (219, 220).

The acidic pathway starts with (25R)26-hydroxylation and (25R)26-carboxylation of cholesterol by CYP27A1 and this and many other steps may proceed extrahepatically (**Figure 8**). Most of the enzymes involved in the neutral pathway are also involved in the acidic pathway although not in the same order (361). An exception is CYP7A1, which is replaced by CYP7B1 as the 7α-hydroxylase in the acidic pathway. CYP7B1 is expressed in many tissues, not just liver (362, 363), and unlike CYP7A1 uses side-chain oxysterols as its substrate. The acidic pathway mostly generates chenodeoxycholic acid rather than cholic acid, so CYP8B1 has minor involvement. The order of A-ring reduction and side-chain cleavage can be reversed in the acidic pathway with the formation of bile acid intermediates possessing 3-oxo-4-ene or 3β-hydroxy-5-ene functions.

#### Steroid Hormone Biosynthesis

The classical steroid hormones aldosterone (mineralocorticoid), cortisol (glucocorticoid), testosterone (male sex hormone) and 17β-estradiol (female sex hormone) are all formed from cholesterol through multiple oxidation reactions (**Figure 9**) (364, 365). Pregnenolone formed *via* CYP11A1 oxidation of cholesterol represents an intermediate between oxysterol and steroid hormone biosynthesis. It is oxidized by HSD3B2 to progesterone on the pathway to aldosterone or by CYP17A1 to 17α-hydroxypregnenolone on the route to cortisol. Both these pathways use CYP21A2 as a C-21 hydroxylase and CYP11B enzymes as the 11β-hydroxylase. Note, HSD3B1 is the enzyme which generates progesterone from pregnenolone in the placenta, when progesterone acts as the hormone of pregnancy. Further oxidation of 17α-hydroxypregnenolone by CYP17A1 leads to dehydroepiandrosterone on the road to testosterone and17β-estradiol. In this pathway additional oxidations and reductions are carried out by HSD3B2 and HSD17B3, respectively, while CYP19A1 is required to generate estrogens. Besides being synthesized in the adrenal gland and sex organs it is noteworthy that steroids can be synthesized in the brain and are then named neurosteroids (366, 367). A more general term for brain steroids which may be synthesized in the brain or imported from the periphery and exert rapid non-genomic effects is neuroactive steroids. It is beyond the scope of this review to discuss steroid hormone biosynthesis and metabolism in greater detail and the reader is directed to the excellent reviews of Shackleton and colleagues (364, 368–370).

## Concluding Remarks

Enzymatically oxidized lipids are derivatives of PUFA or cholesterol with critical functions in cellular and physiological processes as signaling mediators and hormones. Their biosynthesis is highly regulated and carried out by enzymes that include LOXs, COXs, CYPs, and AKRs. Advances in our understanding of these enzymes have led to the discovery of novel lipid mediators and their biosynthetic routes. Many functional aspects of these enzymes and their products remain unclear, requiring further investigation. These include elucidating the function of 15-LOX-2 (ALOX15B) in macrophages, the regulatory mechanisms of 12-LOXs and eLOX3, the mechanistic details of transcellular biosynthesis, the origin of 18-HEPE required for E-resolvin biosynthesis, the biological functions of orphan CYPs, and the bioactivities/functions of oxygenated endocannabinoids. The relative physiological importance of some multiply oxygenated PUFA mediators, which are generated in extremely low amounts, also needs to be further clarified. This is also true for multiply oxygenated derivatives of cholesterol.

Research on oxygenated PUFA and oxysterols has been carried out largely in parallel, and both areas have benefited greatly from advances in analytical methods. However, there are still major questions to be answered in terms of their proposed roles in human disease including atherosclerosis and neurodegeneration. Here, we presented biosynthetic pathways of oxygenated PUFA and oxysterols, highlighting their (known) functions to show the diversity of products but also to draw connections between the two groups. An integrated approach encompassing the analysis of both lipid groups could be useful in examining the etiology of disease. Despite their involvement in the progression of disease such as atherosclerosis and neurodegenerative disease, the two lipid groups are rarely analyzed together. We propose that oxygenated PUFA and oxysterols are more connected than previously thought, especially as 15-LOX is being increasingly recognized as a regulator of cholesterol metabolism. The immune and nervous systems are both of particular interest as major sites of cholesterol metabolism and 15-LOX expression. Further work on the enzymology of 15-LOX with cholesteryl substrates could lead to the discovery of novel oxidized lipids. For example, 15-LOX oxygenation of PUFA esters of oxysterols could generate novel lipids that link both classes directly in cell types that possess the enzymatic machinery for oxysterol and oxylipin biosyntheses (e.g., macrophages). An alternative biosynthetic route could be through the esterification of oxylipins with free oxysterols. That said, the detection of these proposed molecules could be challenging due to low abundance and sensitivity to alkaline conditions commonly used in analysis. Finally, the substrate promiscuity of many of the enzymes involved in production of oxidized lipids provides a technical challenge for dissection of (patho)physiological function of specific oxidized lipids. Clearly, there is plenty of scope for ongoing exploration of the molecular, cellular, and physiological functions of oxidized lipids.

## Author Contributions

AH planned the manuscript. AH, WG, and YW wrote the manuscript with input from AF and VO. All authors contributed to the article and approved the submitted version.

## Acknowledgments

AAH acknowledges funding from Kuwait University and support from the Kuwait Cultural Office (KCO) in London. Work in Swansea was supported by the UK Biotechnology and Biological Sciences Research Council (BBSRC, grant numbers BB/N015932/1 to WJG and BB/L001942/1 to YW). AJF is supported by funding from Barts Charity. VBO is a Royal Society Wolfson Merit Award Holder and acknowledges funding from the Wellcome Trust for LIPID MAPS (203014/Z/16/Z).

## Conflict of Interest

The authors declare that the research was conducted in the absence of any commercial or financial relationships that could be construed as a potential conflict of interest.

## Abbreviations

4-HNE, 4-hydroxy-2-nonenal; AA, arachidonic acid; AKR, aldo-keto reductase; ALA, α-linolenic acid; COX, cyclooxygenase; CE, cholesteryl ester; CYP, cytochrome P450; DGLA, dihomo-γ-linolenic acid; DHA, docosahexaenoic acid; DPEP2, dipeptidase 2; EET, epoxy-eicosatrienoic acid; EPA, eicosapentaenoic acid; GGT, γ-glutamyl transpeptidase; GLA, γ-linolenic acid; GPCR, G protein-coupled receptor; HETE, hydroxy-eicosatetraenoic acid; HpETE, hydroperoxy-eicosatetraenoic acid; HSD, hydroxysteroid dehydrogenase; LA, linoleic acid; LOX, lipoxygenase; LTA_4_, leukotriene A_4_; lysoPL, lysophospholipid; oxPL, oxidized phospholipids; PG, prostaglandin; PL, phospholipid; PUFA, polyunsaturated fatty acid; SDR, short-chain dehydrogenase/reductase; SPM, specialized pro-resolving mediator.
